# Meiocyte Isolation by INTACT and Meiotic Transcriptome Analysis in *Arabidopsis*

**DOI:** 10.3389/fpls.2021.638051

**Published:** 2021-03-04

**Authors:** Lucia Barra, Pasquale Termolino, Riccardo Aiese Cigliano, Gaetana Cremona, Rosa Paparo, Carmine Lanzillo, Maria Federica Consiglio, Clara Conicella

**Affiliations:** ^1^Institute of Biosciences and Bioresources, National Research Council of Italy, Portici, Italy; ^2^Sequentia Biotech SL, Barcelona, Spain

**Keywords:** meiosis, meiocyte, tagged nuclei, RNA-seq, meiotic transcriptome

## Abstract

Isolation of nuclei tagged in specific cell types (INTACT) is a method developed to isolate cell-type-specific nuclei that are tagged through *in vivo* biotin labeling of a nuclear targeting fusion (NTF) protein. In our work, INTACT was used to capture nuclei of meiocytes and to generate a meiotic transcriptome in *Arabidopsis*. Using the promoter of *AtDMC1* recombinase to label meiotic nuclei, we generated transgenic plants carrying *AtDMC1:NTF* along with biotin ligase enzyme (*BirA*) under the constitutive *ACTIN2* (*ACT2*) promoter. *AtDMC1*-driven expression of biotin-labeled *NTF* allowed us to collect nuclei of meiocytes by streptavidin-coated magnetic beads. The nuclear meiotic transcriptome was obtained by RNA-seq using low-quantity input RNA. Transcripts grouped into different categories according to their expression levels were investigated by gene ontology enrichment analysis (GOEA). The most enriched GO term “DNA demethylation” in mid/high-expression classes suggests that this biological process is particularly relevant to meiosis onset. The majority of genes with established roles in meiosis were distributed in the classes of mid/high and high expression. Meiotic transcriptome was compared with public available transcriptomes from other tissues in Arabidopsis. Bioinformatics analysis by expression network identified a core of more than 1,500 genes related to meiosis landmarks.

## Introduction

Meiosis is a complex process critical to sexual reproduction. In plants, meiosis appears to be influenced by environmental cues (reviewed in De Storme and Geelen, 2014; Si et al., 2015). Within this context, global climate change is expected to have an impact on crop production with consequences for food security (Parry et al., 1999). To face the new challenges, a fundamental understanding of meiosis is required in model, crop, and non-model plants (Lambing and Heckmann, 2018). Molecular knowledge of plant meiosis has primarily advanced through understanding the function of single genes involved in key steps being benefited by the conserved pathways across model species (reviewed in Mercier et al., 2015). Thereafter, transcriptome studies of specific cell types improved our understanding of the gene-expression landscape during meiosis (reviewed in Dubowic-Shulze and Chen, 2014). However, the isolation of plant meiocytes is challenging due to the difficulty in accessing the germline cells. Pollen mother cells and embryo sac mother cells are enclosed by sporophytic tissues, i.e., anthers and ovules inside the flower buds. Over the last decade, different techniques were established for targeted isolation of meiocytes by micromanipulation (Chen et al., 2010; Libeau et al., 2011; Yang et al., 2011; Nelms and Walbot, 2019) and laser microdissection (Tang et al., 2010; Schmidt et al., 2011; Barra et al., 2012; Yuan et al., 2018).

An alternative method that achieves the isolation of nuclei tagged in specific cell types (INTACT) was developed by Deal and Henikoff (2011). INTACT overcomes the limitation of time-consuming manual dissection generally associated with contamination of undesired cells. Therefore, INTACT allows rapid and efficient nucleus isolation with the advantage of not requiring specialized and expensive equipment (Deal and Henikoff, 2010). INTACT is based on *in vivo* biotin labeling of a nuclear targeting fusion (NTF) protein which consists of three parts corresponding to (1) a unique target peptide of biotin ligase recognition (BLRP), (2) a domain from *Arabidopsis* RAN GTPASE ACTIVATING PROTEIN 1 (RanGAP1) for nuclear envelope localization (Rose and Meier, 2001), and (3) green fluorescent protein (GFP) for visualization. BLRP acts as a substrate for the BirA biotin ligase enzyme from *Escherichia coli* (Beckett et al., 1999). *BirA* and *NTF* need to be co-expressed in the same cell.

So far, INTACT has been used in combination with transcriptomic, epigenomic, and proteomic studies in different species including plants (Amin et al., 2014; Agrawal et al., 2019; Del Toro-De León and Köhler, 2019). However, in plants, a broad use of INTACT across different cell types remains challenging since it requires a cell-type marker and a method for genetic transformation for the species of interest. A peculiarity of INTACT is that it provides nuclear RNAs which could differ from cytosolic RNAs depending on the selective compartment enrichment of RNAs influenced by mechanisms such as nuclear retention and posttranscriptional regulation (Reynoso et al., 2018). For instance, the comparison between nuclear and total RNAs furnished additional insights into the transcriptome regulation in the early Arabidopsis embryo (Palovaara et al., 2017). In the future, INTACT could contribute to the elucidation of the transcriptome reorganization occurring in meiosis, particularly at the prophase I expression transitions that were recently defined in maize by single-cell RNA sequencing (Nelms and Walbot, 2019).

In this work, we applied the INTACT-based approach to obtain purified meiocyte nuclei from the total cell pool of floral bud in *Arabidopsis thaliana*. To label meiotic nuclei, we used the promoter of *AtDMC1* recombinase (Klimyuk and Jones, 1997; De Muyt et al., 2009). The AtDMC1-driven expression of biotin-labeled NTF allowed us to isolate meiocyte nuclei using streptavidin-coated magnetic beads. The meiotic nuclear transcriptome was obtained by RNA-seq and validated through analysis of the expression of known meiotic genes and the comparison with other tissues. Finally, expression network analysis was performed to find new candidate genes involved in the meiotic process.

## Materials and Methods

### Plant Material, Transformation, and Growth Conditions

The transgenic line expressing *ACT2:BirA*, kindly given by Prof. R. Deal (Emory University, United States), and the reference ecotype Col-0 of *A. thaliana* (NASC stock N60000) were used in this work. The transgenic line was transformed by *Agrobacterium tumefaciens* (GV3101) according to floral dip method (Clough and Bent, 1998) to obtain plants carrying *DMC1:NTF* along with ACT2:BirA. About 1,500 seeds obtained after transformation were sterilized with 70% ethanol for 1 min., and bleach solution (10% commercial bleach and 0.05% Tween 20) for 10 min., finally washed with sterile water three times. Sterilized seeds were sown on a selective modified MS (Murashige and Skoog, 1962) medium containing hygromycin (50 mg/l) and 0.8% agar, kept for 2–3 days at 4°C, and germinated under long-day conditions (16 h light/8 h darkness) at 24°C. Seedlings transferred to pots were grown in a controlled growth chamber under long-day conditions. The A*DMC1:NTF/ACT2:BirA* double homozygous transgenic line (D.1) was selected from ten T_2_ independent transformant lines. About 200 plants from the D.1 line were grown as above reported for subsequent experiments.

### PCR and RT-qPCR

To confirm transgene insertion, PCR was conducted on the hygromycin resistance gene used as a selectable marker. Genomic DNA was extracted from leaf tissue of transgenic plants using the DNeasy Plant Mini Kit (Qiagen)^1^ according to the manufacturer’s instructions. PCR reactions were performed on genomic DNA using primers listed in Supplementary Table 1. RT-qPCR was performed to verify expression of target *BirA* gene under the constitutive promoter ACT2 in the floral buds. Total RNA from transgenic *ACT2:BirA* plants was extracted using RNeasy plant minikit (QIAGEN) following the manufacturer’s instructions. mRNA was retrotranscribed to cDNA using Superscript III reverse transcriptase (Thermo Fisher Scientific, United States) and oligo dT(20) following the manufacturer’s conditions, and relative expression was verified by real-time qPCR. The experiment was performed on a QIAGEN Rotorgene 6,000 qRT-PCR machine, using Power Sybr Green real time mix (Thermo Fisher Scientific, United States). The reaction conditions were as follows: one cycle at 95°C for 10 min, and 40 cycles of 95°C × 10″ denaturation and 60°C × 45″ annealing and extension. The melting curve was run to verify the specificity of the primers. The *ADENINE PHOSPHORIBOSYL TRANSFERASE 1* (*APT1*) gene was used as a housekeeping internal control. Target and housekeeping primers are listed in Supplementary Table 2.

### Construct for INTACT, Nucleus Isolation, and Microscopy

The fusion gene *NTF* carried in the vector ADF8-NTF, kindly given by Prof. R. Deal (Emory University, United States), and the *AtDMC1* promoter carried in the vector SLJ7753, kindly given by Prof. J.D.G. Jones (The Sainsbury Laboratory, Norwich, United Kingdom), were amplified with specific primers carrying GATEWAY adapters. Subsequently, they were cloned into Gateway vectors pDONR/Zeo and pDONR/P4P1R, respectively (Thermo Fisher Scientific). Generated entry clones were recombined with multisite GATEWAY reaction into destination vector pH7m24GW,3^2^, and the final expression clone, named pEXPR-DMC1-NTF, was generated. All gateway reactions were performed following the manufacturer’s standard protocols. Primers used for vector construction are listed in Supplementary Table 3.

Flower buds of the size corresponding to male meiotic stage (Smyth et al., 1990) were collected, fixed with 1% formaldehyde solution, and stored at −80°C. Nuclei were purified from 1.5 g of frozen and homogenized tissue as described previously by Wang and Deal (2015) with some minor modifications. In particular, the tissue immersed in 40 ml of Nuclei Purification Buffer solution (NPBf) containing 20 mM MOPS (pH 7), 40 mM NaCl, 90 mM KCl, 2 mM EDTA, 0.5 mM EGTA, 0.5 mM spermidine, 0.2 mM spermine, 1% (v/v) formaldehyde was incubated in 50 ml tube under vacuum glass desiccator for 10 min., and vacuum release for 2 min. The procedure was repeated once. Then, glycine was added to a final concentration of 0.125 M under vacuum for 7 min. After NPBf solution elimination, the tissue was washed three times with water. Isolated nuclei were resuspended into RNAlater buffer, and RNA extraction was performed immediately.

Laser-scanning confocal microscopy imaging was performed using a confocal Zeiss LSM 510. Isolated nuclei were observed using a Florescence Microscope (Leitz Aristoplan).

### RNA Extraction and Sequencing

Total RNA was extracted from three replicates of isolated nuclei using an RNeasy Extraction Mini Kit (Qiagen, see text footnote 1) according to the manufacturer’s instructions. Isolated nuclei derived from the same population of inflorescences where the buds different from stage 9 (Smyth et al., 1990) had been manually dissected. Quantification was performed on a QUBIT fluorometer.

RNA sequencing was performed by IGA Technology Services Srl^3^. The libraries were produced using retrotranscribed cDNA previously amplified by Ovation Ultralow Library System V2 (NuGEN Technologies, Inc.). Library size and integrity were assessed using the Agilent Bioanalyzer (Santa Clara, CA) or Caliper GX (PerkinElmer, MA). Sequencing was performed by Illumina HiSeq 2,500 (Illumina, San Diego, CA) and 30-M paired-end reads (2 × 125) per replicate were generated.

### Bioinformatics

Raw sequencing data (FASTQ files) were quality checked using the software FASTQC v0.11.5^4^, then low-quality bases and adapter sequences were removed with the software BBDuk v35 (Bushnell et al., 2017) setting 35 bp as the minimum read length after trimming. The read length ranged from 35 to 125 bp after trimming. More than 98% of the final reads had a length of 125 bp since adapter contamination was very low. The trimmed reads were then mapped against the *A. thaliana* reference genome (Araport11) (Cheng et al., 2016) using STAR v2.7.3a with the option – alignEndsProtrude 100 DiscordantPair. The statistics from the mapping step were produced with Qualimap v2.2.1 (Okonechnikov et al., 2016). Kallisto v0.46.0 was used to obtain transcript expression quantification levels, as estimated counts and TPMs, which were then summarized as gene expression values using the R package “tximport” (Soneson et al., 2016).

The correlation analysis of the samples was performed using the R in-built function “cor,” and the results were plotted with the function “corrplot.” The classification of the gene classes and the saturation analysis were performed with the R package “NOISeq” (Tarazona et al., 2015). Genes were classified based on their average expression levels using the following criteria: “undetected” if the expression was 0 across all the replicates, “Low” if log2 average TPM ≤ 1.252, “Low_Mid” if log2 average TPM > 1.252 and ≤ 2.207, “Mid_High” if log2 average TPM > 2.207 and ≤ 4.169, and “High” if log2 average TPM > 4.169. The abovementioned values correspond to the 25th, 50th, and 75th percentiles of the log2 average TPM distributions, respectively.

In order to compare the expression profile of the meiocytes against other datasets, TPM values from the following datasets were downloaded from EBI: E-GEOD-38612, E-GEOD-55866, and E-MTAB-4202 (Supplementary Table 4). In addition, gene expression profiles from pollen and tapetum cells were obtained from Loraine et al. (2013) and Li et al. (2017). When multiple replicates of the same tissue were available, an average was calculated. Similarly, when multiple developmental stages of the same tissue were available, the average was calculated. The only exception consisted of the tapetum datasets kept separate because the two available stages were highly different. Finally, a complete expression matrix was obtained by merging all the datasets and the ARSyN function from the NOISeq package was used to remove the batch effect. A PCA analysis was then performed with the in-built “prcomp” function in R and the results plotted with “ggplot2.”

A gene expression network was generated using the expression matrix obtained after the ARSyN correction, following the Aracne algorithm implemented in the R package “parmigene” (Sales and Romualdi, 2011). Cytoscape v3.8.1 was then used to plot the network and select only the first neighbors of known meiotic genes in order to find new candidates. The gene ontology (GO) analysis of the obtained genes was then performed using the Cytoscape app named “BinGO” using the hypergeometric test as a statistical test and an FDR lower than 0.05 as threshold. All the heat maps were generated using Clustvis (Metsalu and Vilo, 2015).

Gene ontology enrichment analysis (GOEA) were performed using in-house scripts based on the method described in Tian et al. (2017) and setting a minimum FDR threshold of 0.05.

## Results

### Generation of Transgenic Material and Isolation of Nuclei From Meiocytes Using INTACT in Arabidopsis

**FIGURE 1:**
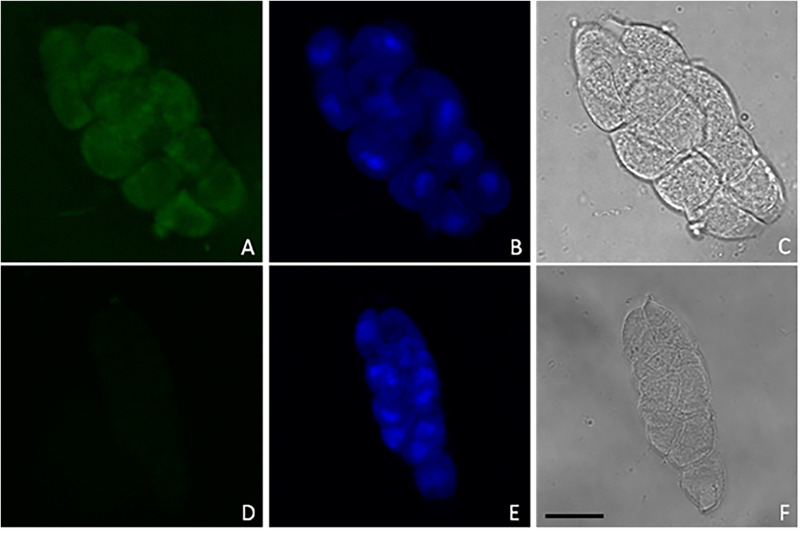
Representative confocal images of GFP **(A,D)** and DAPI **(B,E)** fluorescence in male meiocytes along with phase contrast observations **(C,F)** from *AtDMC1*:*NTF/ACT2*:*BirA* line **(A–C)** and wild type **(D–F)**. Nuclei (blue) were stained by DAPI (10 μg/ml) to visualize DNA. Scale bar: 10 μm.

**FIGURE 2:**
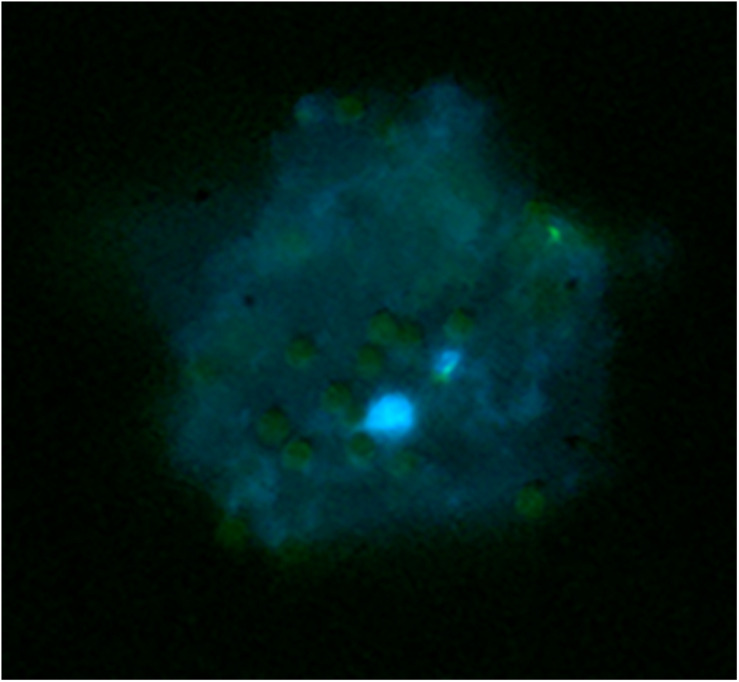
Capture of biotinylated nuclei from meiocytes in the *AtDMC1*:*NTF/ACT2*:*BirA* line using streptavidin-coated magnetic beads. Nuclei (blue) stained with DAPI are surrounded by 2–8-μm spherical beads (pale green).

In order to isolate meiocyte nuclei by the INTACT method for RNA-seq analysis in Arabidopsis, we generated transgenic material carrying a *NTF* protein under the meiosis-specific *AtDMC1* promoter (Klimyuk and Jones, 1997) along with the biotinilase *BirA* under a constitutive *ACTIN2* (*ACT2*) promoter. The promoter of *ACT2* (At3g18780) was used by Deal and Henikoff (2011) to drive expression of *BirA* in root cell types (An et al., 1996). Following detection of *ACT2:BirA* expression in floral buds (not shown), we used the same transgenic line generated by Deal and Henikoff (2011) as starting material. In this line, we introduced the new construct carrying the *AtDMC1:NTF* expression cassette. Although *AtDMC1* (At3g22880) was expected to drive *NTF* expression in both male and female meiocytes (Klimyuk and Jones, 1997), we restricted our confocal microscopy analysis to anthers and male meiocytes. Indeed, the flower buds were collected at a stage corresponding to male meiosis which occurs earlier than female meiosis (Schneitz et al., 1995). Confocal microscopic examination of the *AtDMC1*:*NTF/ACT2*:*BirA* line showed that *NTF* was expressed in the expected cell type. Indeed, we detected GFP-positive male meiocytes during prophase I stage, arguably before the nuclear envelope breakdown (NEBD; Figure 1). To purify labeled nuclei from meiocytes, we extracted total nuclei from whole inflorescences with buds at floral stage 9 (Smyth et al., 1990) in the *AtDMC1*:*NTF/ACT2*:*BirA* line. Then, the nuclei were incubated with streptavidin-coated magnetic beads according to INTACT protocols (Deal and Henikoff, 2011; Wang and Deal, 2015). After nucleus isolation, we used a fluorescence microscope to observe the complex DAPI-stained nuclei/beads (Figure 2).

### RNA-seq From Isolated Nuclei of the Meiocytes

**FIGURE 3:**
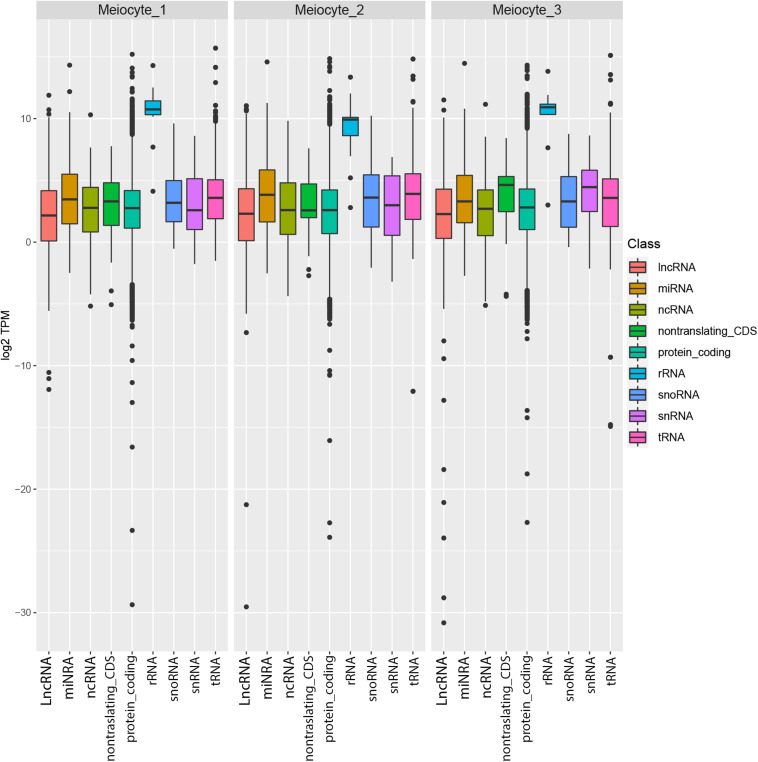
Distribution of the expression levels of *Arabidopsis thaliana* genes in the meiocytes. Genes are divided in multiple biotypes according to the official annotation, and for each one the distribution of log2 TPM values is represented as box plots.

To obtain the meiotic nuclear transcriptome, RNA sequencing (RNA-seq) was performed downstream of INTACT. RNA extracted from isolated nuclei of meiocytes in three replicates was sequenced by using the Illumina technology following an amplification step. Pearson’s correlation coefficient indicated the reliability of the experiment (Supplementary Figure 1). After trimming, an average of 85,927,325 reads per replicate were obtained (Supplementary Table 5). About 77% of these reads were mapped onto the Arabidopsis genome (Supplementary Table 6). The percentages (calculated as average of the replicates) of the uniquely mapped reads located in known exons, introns, and intergenic regions are 38, 26, and 36%, respectively (Supplementary Figure 2). Given the high percentage of reads that mapped to multiple positions (Supplementary Table 6), the gene expression, measured as TPM (Transcript Per Million), was calculated by a specific algorithm (Kallisto) which is able to process multiple mapping reads (Supplementary Table 7). A frequency ranging between 75.2 and 81.3% of the genes (total no. 32,833) was detected across the replicates (Supplementary Figure 3). Transcript types of each replicate are summarized in Figure 3 showing the distribution of expression profiles for the different gene classes. Ribosomal RNA appeared the most abundant class whereas lncRNA is the less expressed class. This result is consistent with a recent study in barley in which 65% of the downregulated DEGs were lncRNAs in leptotene/zygotene vs. pre-meiosis comparison (Barakate et al., 2020).

**FIGURE 4:**
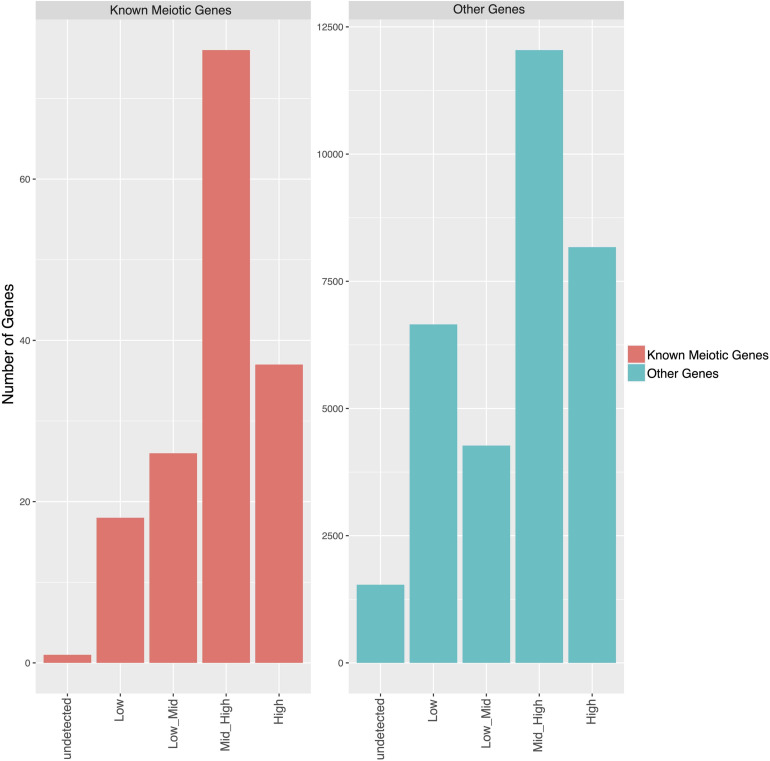
Bar plot showing the number of genes classified in expression ranges in the meiotic cells. Genes were classified based on their average expression levels using the following criteria: “undetected” if the expression level was 0 in all the replicates, “Low” if log2 average TPM ≤ 1.252, “Low_Mid” if log2 average TPM > 1.252 and ≤ 2.207, “Mid_High” if log2 average TPM > 2.207 and ≤ 4.169, and “High” if log2 average TPM > 4.169. Genes were divided into known meiotic genes (left) and other genes (right).

Transcripts were grouped into four classes according to their expression levels from low to high (Supplementary Figure 4). GOEA was performed to identify enriched (i.e., over-represented) GO terms associated with the different expression classes (Supplementary Figures 5A–D). The most enriched GO terms, such as “DNA demethylation” in the mid/high-expression class and “RNA stabilization” in the high expression class, suggest that these biological processes are particularly relevant to meiosis. To assess the reliability of our results, we surveyed the genes with documented functions in Arabidopsis meiosis. A list of 197 meiotic genes was implemented using GO terms GO:0051321 (meiotic cell cycle) and GO:0140013 (meiotic nuclear division) (Supplementary Table 8). The majority of meiotic genes were distributed in the classes of mid/high and high expression while a small number were in the low-expression class (Figure 4). By comparison, this class was the third most represented for the total of transcripts (Supplementary Figure 4).

### Transcriptome Comparison Between Male Meiocytes and Other Tissues

To further characterize our meiotic transcriptome, we compared it with publicly available transcriptomic data from other specific tissues and cell types of Arabidopsis. Initially, we planned to compare our RNA-seq data with those from male meiocytes isolated by micromanipulation in Arabidopsis (Chen et al., 2010; Yang et al., 2011) but, unfortunately, these datasets are not available.

**FIGURE 5:**
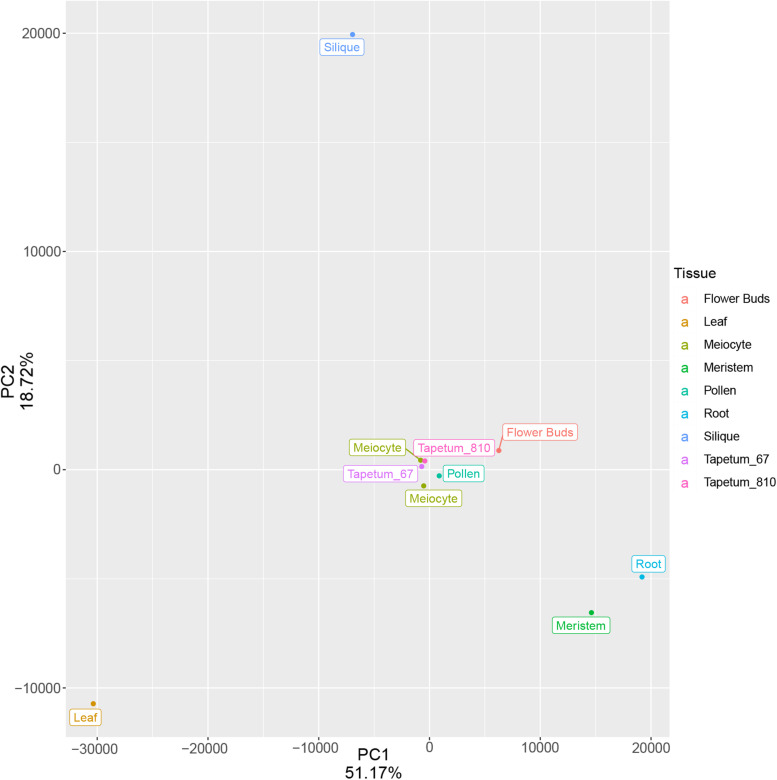
Principal component analysis of meiocytes (current study) and multiple Arabidopsis tissues after batch correction of the TPM values. The variance associated with each PC is shown in the plot. Each point represents a tissue/cell type.

**FIGURE 6:**
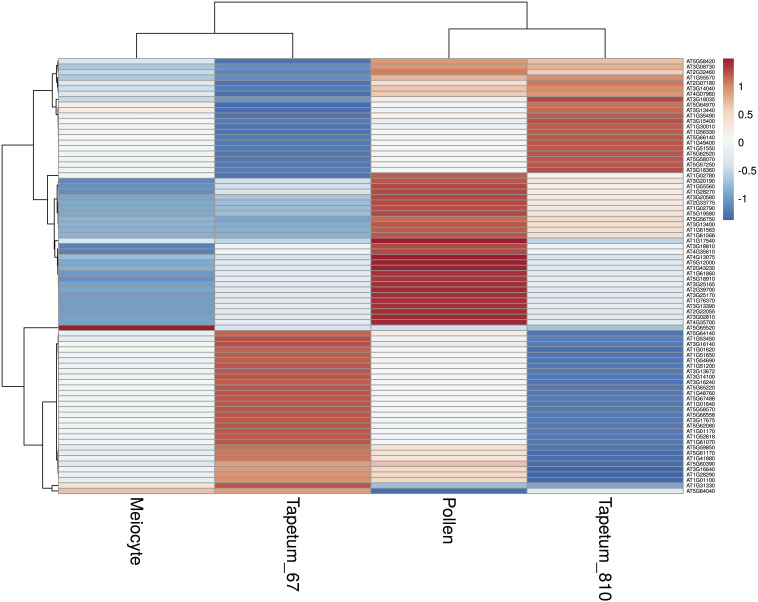
Heat map showing the expression profile of genes considered to be pollen or tapetum specific. Each line represents a gene; the gradient of color was obtained after scaling of the TPM values and after batch correction of the expression data. The meiocyte data corresponds to the average of the two replicates. The genes and the samples are grouped applying a hierarchical clusterization on the Pearson correlation values as shown by the hierarchical tree on the left and on the top.

Based on the low expression of *AtDMC1* (Supplementary Figure 6), we discarded replicate 1 in all the subsequent analyses. Principal component analysis (PCA) performed with meiocytes (current study), meristem, leaf, root, flower buds, tapetum (from two different developmental stages), pollen, and silique revealed that meiocytes clustered close to flower buds, as expected, but also to tapetum and pollen (Figure 5). We evaluated the expression profile of genes (list reported in Supplementary Table 9) considered to be specific for these two types of cells (Loraine et al., 2013; Li et al., 2017). The heat map revealed expression patterns specific for meiocytes, tapetum, and pollen (Figure 6). On the other hand, a hierarchical sample dendrogram showed that meiocyte sample groups together with tapetum 6–7 as well as pollen with tapetum 8–10 equivalent to a later stage.

**FIGURE 7:**
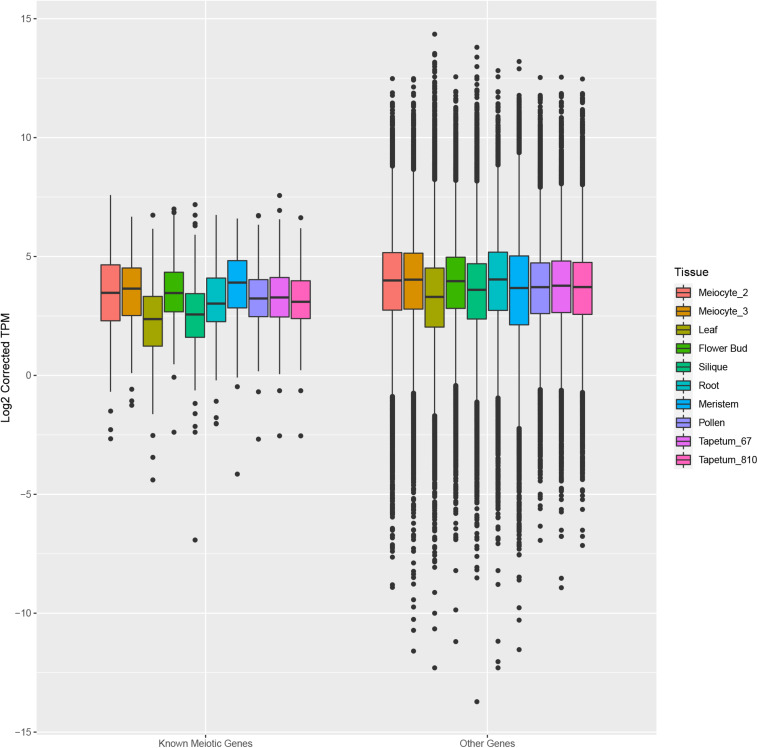
Box plots showing the distribution of expression values of known meiotic genes (left) and other genes (right) in meiocytes (current study) and multiple Arabidopsis tissues. The box corresponds to the interquartile range (IQR), and the black line inside each box represents the median. The upper whisker extends from the hinge to the largest value no further than 1.5 * IQR from the hinge. The lower whisker extends from the hinge to the smallest value at most 1.5 * IQR of the hinge. Data beyond the end of the whiskers are called “outlying” points and are plotted individually.

**FIGURE 8:**
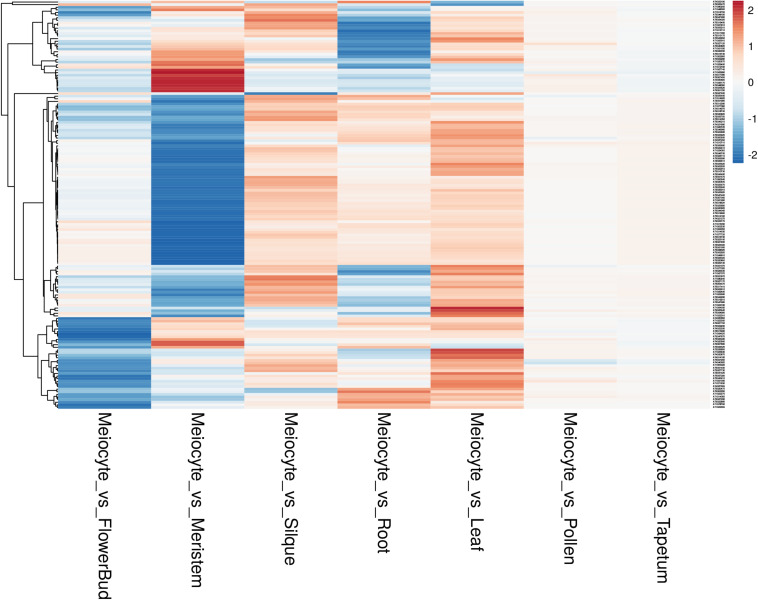
Heat map showing the gene expression fold changes between meiocytes and multiple Arabidopsis tissues. Each line represents a gene; the gradient of color was obtained after scaling of the log2 fold changes and after batch correction of the expression data. The genes are grouped applying a hierarchical clustering on the Pearson correlation values as shown by the hierarchical tree on the left.

Afterward, we assessed the overall gene expression of meiotic genes (list reported in Supplementary Table 8) and of the other transcripts in meiocytes and multiple tissues (Figure 7). The analysis revealed that the median expression value of meiotic genes was higher in isolated meiocytes when compared to the other tissues/cell types with the exception of floral bud and meristem samples. This result is not surprising since the meiocytes are included in floral buds and they share cell-cycle genes with the meristem. The median expression value of the other transcripts was basically steady across the different tissues (Figure 7). Considering the meiotic gene expression fold changes between meiocytes and the other tissues/cell types, the heat map revealed a pattern in agreement with the above reported analysis (Figure 8 and Supplementary Table 10).

### Meiotic Gene Network

**FIGURE 9:**
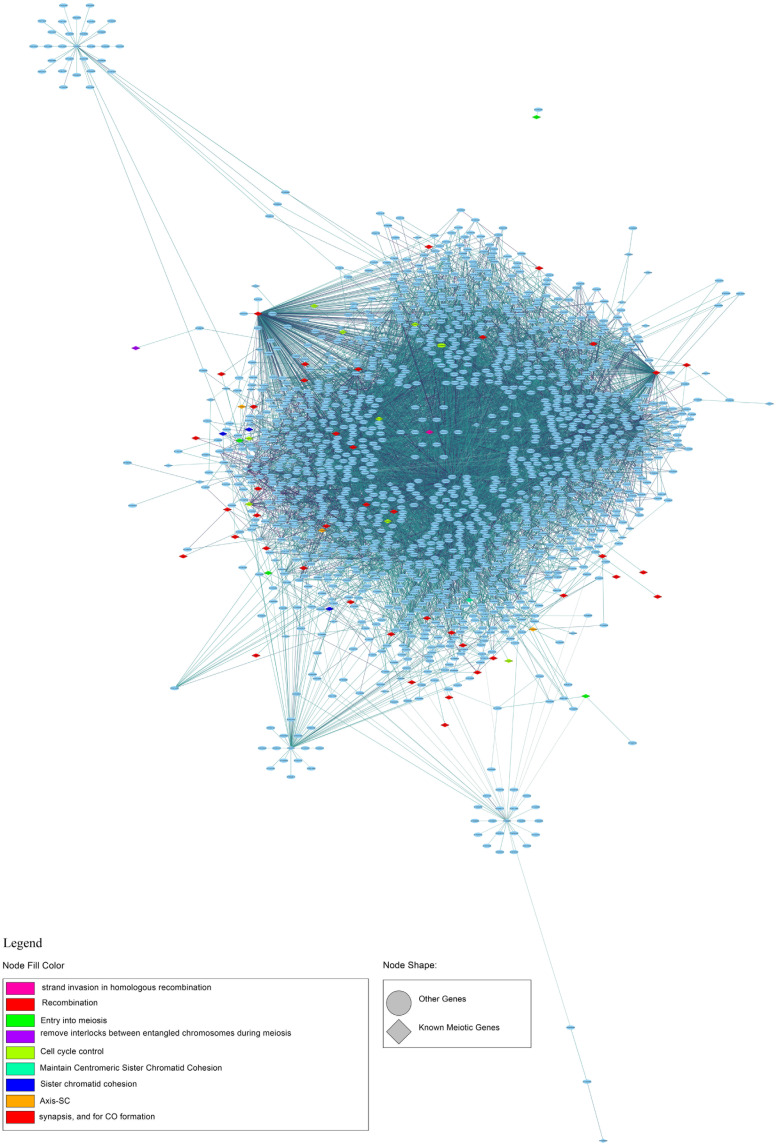
First-neighbor meiotic gene network of co-expression. The legend shows the node fill colors associated with a different meiotic function and the node shape: rhomboidal shape for known meiotic gene and ellipsoidal shape for other genes.

To identify new candidate genes with a meiotic function, we generated a gene expression network based on the genes known to be involved in meiosis (Supplementary Table 8). By selecting the first neighbors of the known meiotic genes, we found 1,503 genes with a total of 7,607 connections (Figure 9 and Supplementary Table 11). The network is characterized by a very large cluster, thereby suggesting that the known meiotic genes and their neighbors are all highly connected as also indicated by an average degree of 4.95 connections. The most connected genes with 435 and 323 connections have a documented role in meiosis. In particular, FIDGETIN-LIKE-1 INTERACTING PROTEIN (FLIP, AT1G04650) forms a protein complex with FIDGETIN-LIKE-1 (FIGL1) that is conserved from Arabidopsis to human, and it regulates meiotic crossover formation via RAD51 and DMC1 (Fernandes et al., 2018). The other gene, *AXR1* (AT1G05180), is involved in the neddylation/rubylation protein modification pathway and TE methylation in meiocytes (Jahns et al., 2014; Christophorou et al., 2020). AXR1 plays a significant role in DNA repair (Martinez-Garcia et al., 2020). Collectively, this finding reinforces the reliability of the analysis performed in this study. New candidates which could play a role in meiosis are two transcription factors (TFs), AT1G06070 and AT1G02220, with 64 and 30 connections, respectively. These two genes belong to the bZIP and NAC TF families, and their function in meiosis has not been documented. Although with less connections than the genes reported above, AT1G04200, described as Dymeclin (DYM, Dyggve–Melchior–Clausen syndrome protein), is an interesting candidate for a possible meiotic function. Indeed, the mouse DYM homolog has a high expression in testis (Yue et al., 2014) and has a proved interaction with FANCD2, a component of the Fanconi anemia DNA repair pathway (Zhang et al., 2017).

**FIGURE 10:**
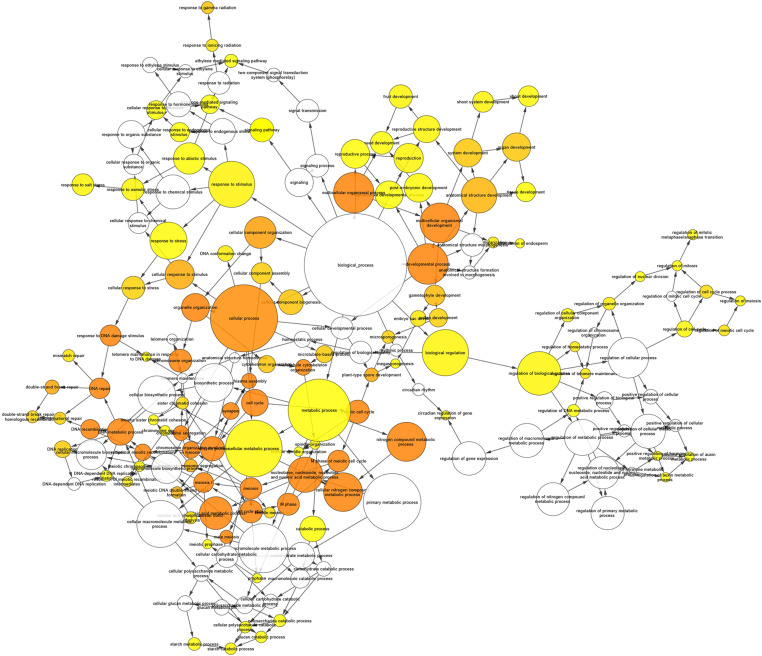
Gene ontology (GO) Enrichment Network of the first neighbors of known meiotic genes obtained by Cytoscape’s BinGO. Each node represents a GO category. The size of the node is proportional to the number of genes in the corresponding GO category. The color of the nodes from yellow to orange is associated with increasing level of significance in the enrichment while white nodes mean no significance.

A GO analysis performed using the genes identified by the network analysis revealed GO targets associated with meiotic processes and, particularly, to Meiosis I such as recombination, synapsis, chiasma assembly, and meiotic chromosome segregation (Figure 10 and Supplementary Table 12).

## Discussion

In this work, we reported the application of the INTACT method in meiocyte isolation in *A. thaliana*. INTACT entails the use of a transgene that can be driven by spatially and/or temporally regulated promoters enabling the isolation of nuclei from specific cell types (Deal and Henikoff, 2010). In our experiment, because of a limited number of effective meiotically active promoters available, we employed the established *AtDMC1* promoter widely used in meiotic studies (Li et al., 2012). AtDMC1 is a recombinase involved in Double Strand Break (DSB) repair and expressed during early prophase I in both male and female meiotic cells in anthers and carpels, respectively (Klimyuk and Jones, 1997; De Muyt et al., 2009). However, AtDMC1 activity is not restricted only to meiotic cells. Indeed, AtDMC1 has been observed in embryonic cell culture (Doutriaux et al., 1998) and in young seedlings (Orel et al., 2003). On the other hand, *AtDMC1* was not expressed in the meiocyte’s neighboring somatic cells (Klimyuk and Jones, 1997; Li et al., 2012). Our analysis has been restricted to male meiocytes collected from floral buds at a stage corresponding to microsporogenesis (Smyth et al., 1990). However, it cannot be excluded that female meiocytes also occur in our sample.

Isolation of nuclei tagged in specific cell types allowed us to obtain a nuclear meiotic transcriptome from meiocytes of *A. thaliana* based on RNA-seq data. On average, we detected the expression of about 25,000 genes corresponding to 76% of total genes. Similarly, approximately 22,000 and 24,000 genes were found to be expressed in Arabidopsis male meiocytes isolated in previous studies based on RNA-seq (Chen et al., 2010; Yang et al., 2011). Consistently, 60% or more of all genes in the genome were estimated to be expressed in rice and Arabidopsis male meiocytes (Schmidt et al., 2012). In Arabidopsis, the male meiotic transcriptome shows the largest overlap (approximately 67%) with tissues having cells in active division, including floral buds, anthers, and shoot apex (Yang et al., 2011). The number of reads in our RNA-seq experiment, and particularly the number of uniquely mapped reads, was higher in our study compared to that previously published (56 vs. 33%) (Yang et al., 2011). In addition, we applied a specific algorithm (Kallisto) to multiple mapping reads (17% on average), thereby quantifying the expression of the genes more precisely. In our experiment, we observed an unexpected proportion of unmapped reads (23% on average) that was likely caused by the library preparation kit for RNA-seq using low-quantity input RNA. Indeed, a performance evaluation study of five methods evidenced that Ovation RNA-seq System V2 from NuGEN (the kit used in our study) generated fewer raw/mapped reads (Song et al., 2018).

Importantly, a distinct feature of our dataset compared to those reported in previous studies (Chen et al., 2010; Yang et al., 2011) is that INTACT provides RNAs occurring within the nucleoplasm. A strong correlation between nuclear RNA and total mRNA was reported in Arabidopsis embryo and endosperm (Palovaara et al., 2017; Del Toro-De León and Köhler, 2019). However, in our study, the comparison with total mRNAs from previous RNA-seq experiments in male meiocytes (Chen et al., 2010; Yang et al., 2011) was prevented due to the unavailability of latter datasets.

Meiosis involves two rounds of cell divisions and the nuclear envelope (NE) undergoes breakdown (NEBD) and reformation twice (Pradillo et al., 2019). In particular, the NEBD occurs at late prophases I and II. Since INTACT requires a NTF transgene which carries a domain for NE localization (Deal and Henikoff, 2010), INTACT does not operate during NEBD. On the other hand, the expression pattern and immunolocalization of AtDMC1 (Klimyuk and Jones, 1997; De Muyt et al., 2009) point out a potential early prophase I-specificity of AtDMC1. For these reasons, we expected the isolated meiocytes to provide a subset of meiotic genes enriched in processes related to early prophase I substages. Apparently, however, the high number of transcripts and the fact that genes involved in later meiotic stages are expressed in our dataset seem to not support our expectations. For instance, the *AtPS1* (*Parallel Spindle 1*, AT1G34355) involved in the orientation of the spindles in Meiosis II (d’Erfurth et al., 2008) was present in the mid-high expression class in our dataset. This finding can be justified by different explanations. First, AtDMC1 could be expressed in other meiotic stages besides prophase I. Second, timing of the gene expression is not strictly associated with timing of the encoded protein function, as evidenced in maize prophase I (Nelms and Walbot, 2019). Finally, the transcriptional basis relevant for meiosis is already set up before its onset, as reported in rice and maize (Tang et al., 2010; Yuan et al., 2018). In the future, other prophase I-expressed genes could be used to build more INTACT lines. For this purpose, we suggest as candidates some meiosis-specific genes with established roles in prophase I identified in mid/high- and high-expression classes of our dataset (Supplementary Table 13). These genes exhibit a very low expression in the other tissues (leaf, root, meristem, flower bud, pollen, tapetum, and silique). In addition, they appear to be expressed also in maize at early prophase I as evidenced by the single-cell RNA-seq experiment (Nelms and Walbot, 2019).

The question whether all genes in meiotic transcriptome are essential for meiosis or whether the large number of transcripts is the result of a global de-repression of chromatin during meiosis remains to be answered (Lambing and Heckmann, 2018). Our study evidenced the prevalence of the biological process “DNA demethylation” when we considered the mid/highly expressed genes. Consistently, the upregulation of transposons (TEs) has been reported to be a prominent feature in Arabidopsis male meiocytes (Chen et al., 2010; Yang et al., 2011), and it supports the suggestion that DNA methylation decreases at meiosis onset (Kawashima and Berger, 2014). Methylome analysis showed that meiocytes have higher CG and CHG methylation but lower CHH methylation in comparison to somatic tissues (Walker et al., 2018). Therefore, it is likely that DNA demethylation engages the CHH context which, on the other hand, is interconnected with TEs. This view is reinforced by the methylome analysis of meiocytes in *axr1* mutant (Christophorou et al., 2020). Indeed, this mutation, which affects general DNA methylation in plants, showed a specific increase in meiocytes for CHH methylation in TEs. Remarkably, gene expression network analysis performed in our work identified *AXR1* as a relevant hub thereby confirming its important roles in meiosis (Jahns et al., 2014; Christophorou et al., 2020; Martinez-Garcia et al., 2020). Furthermore, DNA demethylation could have a profound influence on gene expression through TEs (Wang and Baulcombe, 2020). TEs can act *in cis* to affect expression of adjacent genes as observed in male meiocytes by Yang et al. (2011). Finally, our co-expression network revealed different gene modules related to meiosis as well as novel candidate genes with a potential meiotic role. The functional analysis of the candidate meiotic genes will contribute to our understanding of meiosis.

## Data Availability Statement

Raw sequencing data obtained by the INTACT experiment was deposited in the Sequence Read Archive (SRA) database of NCBI within the BioProject PRJNA668331.

## Author Contributions

LB and PT performed the experiments. PT designed the main experiments. RAC performed the bioinformatics analysis. GC performed the cytological analysis. RP and CL assisted with the experiments. PT, RAC, and GC contributed to the manuscript editing. CC conceived the study and wrote the grant. CC, LB, and FC wrote the manuscript. All authors contributed to the article and approved the submitted version.

## Conflict of Interest

RAC was employed by company Sequentia Biotech SL. The remaining authors declare that the research was conducted in the absence of any commercial or financial relationships that could be construed as a potential conflict of interest.

## Footnotes

1 https://www.qiagen.com/

2 https://gateway.psb.ugent.be/search

3 appliedgenomics.org

4 https://www.bioinformatics.babraham.ac.uk/projects/fastqc/
