# Supplementary material for: Meiocyte Isolation by INTACT and Meiotic Transcriptome Analysis in Arabidopsis
Source: Front Plant Sci. 2021 Mar 4;12:638051. doi: 10.3389/fpls.2021.638051 (PMC7969724; doi:10.3389/fpls.2021.638051)
Supplement: Supplementary file 1 [file Presentation_1.PPTX]

## Slide 1
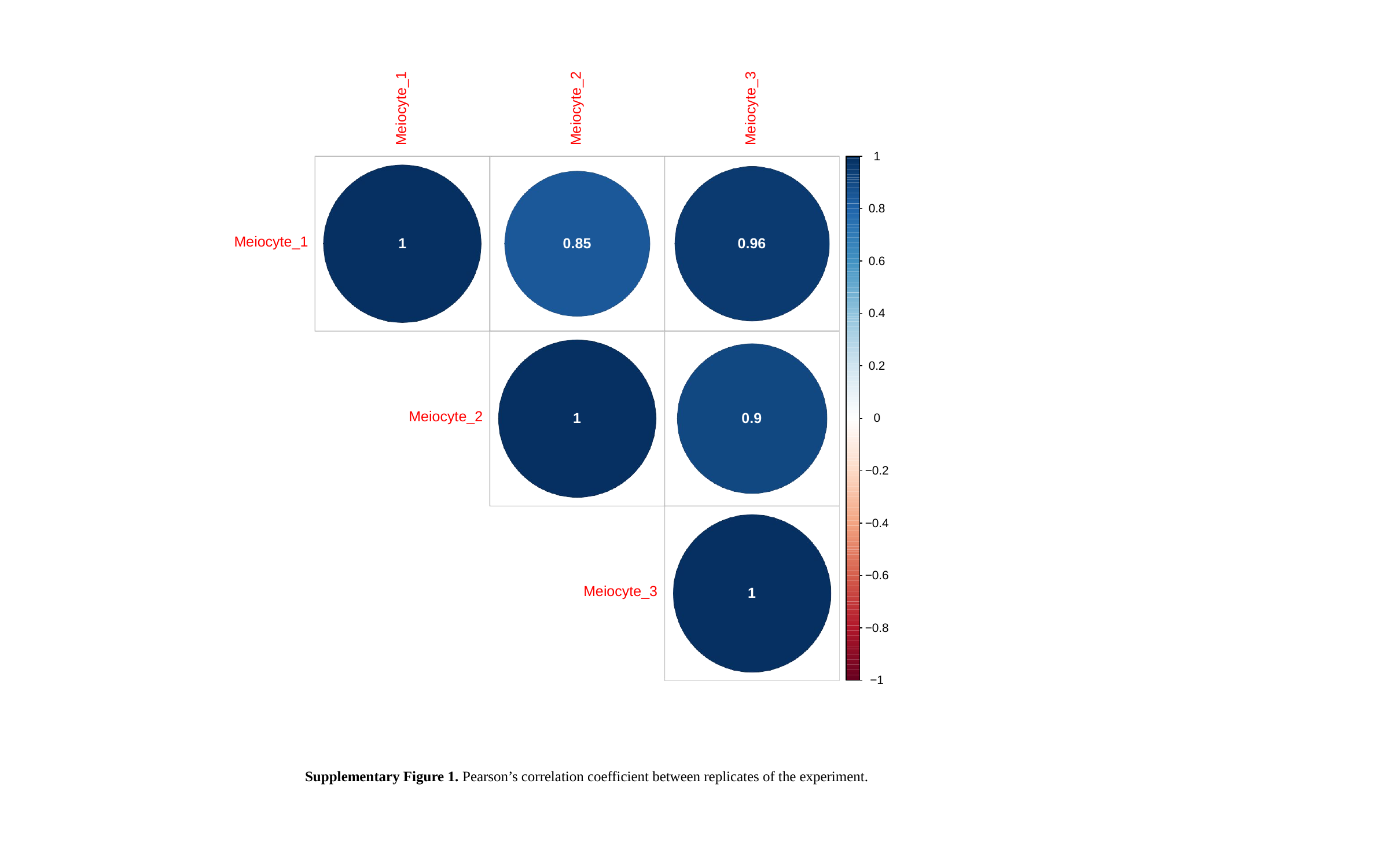

Meiocyte_1
Meiocyte_2
Meiocyte_3
1
0.8
Meiocyte_1
0.85
0.96
1
0.6
0.4
0.2
Meiocyte_2
0.9
1
0
−0.2
−0.4
−0.6
Meiocyte_3
1
−0.8
−1
Supplementary Figure 1. Pearson’s correlation coefficient between replicates of the experiment.
