# Supplementary material for: Meiocyte Isolation by INTACT and Meiotic Transcriptome Analysis in Arabidopsis
Source: Front Plant Sci. 2021 Mar 4;12:638051. doi: 10.3389/fpls.2021.638051 (PMC7969724; doi:10.3389/fpls.2021.638051)
Supplement: Supplementary file 2 [file Presentation_2.PPTX]

## Slide 1
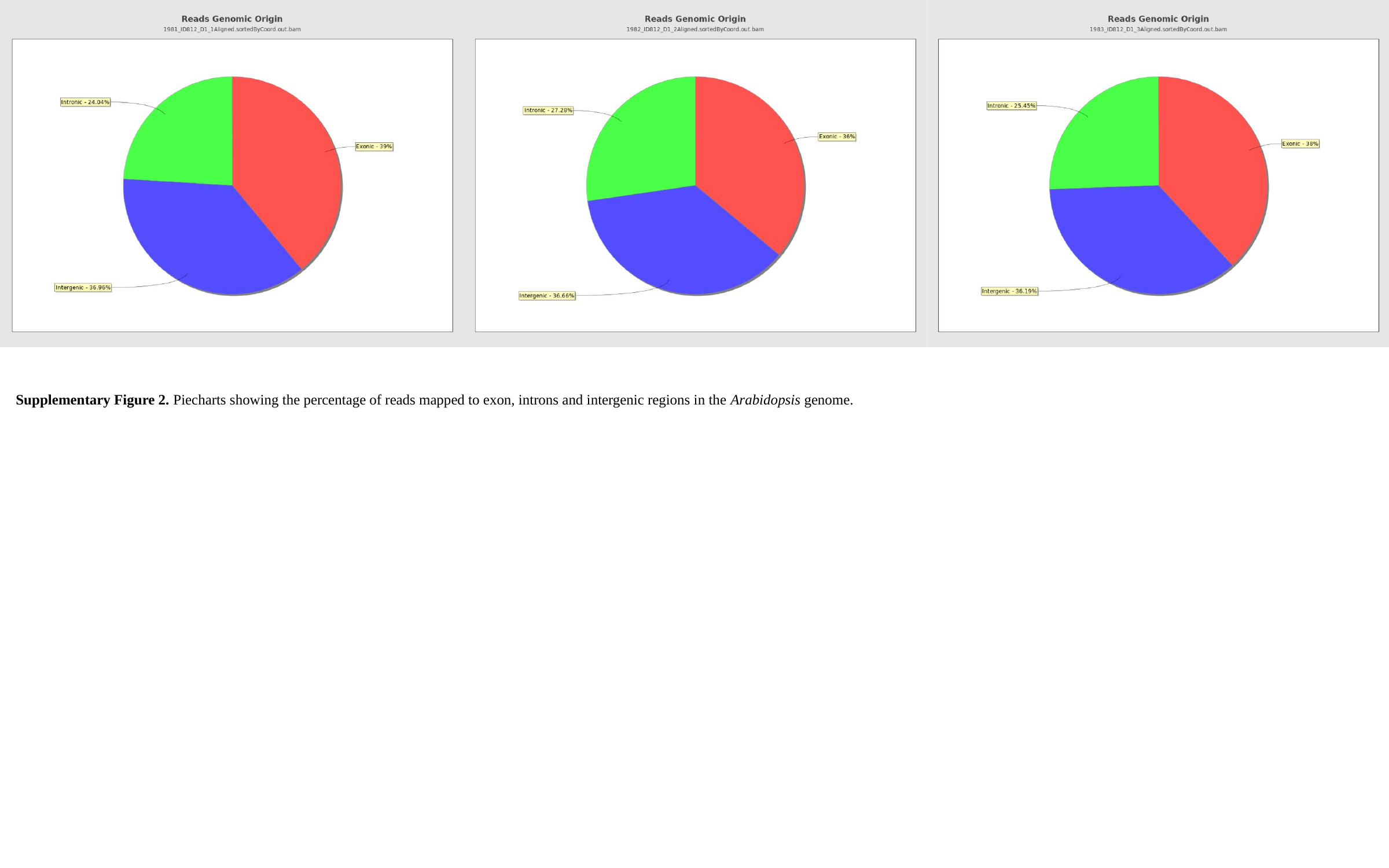

Supplementary Figure 2. Piecharts showing the percentage of reads mapped to exon, introns and intergenic regions in the Arabidopsis genome.
