# Supplementary material for: Meiocyte Isolation by INTACT and Meiotic Transcriptome Analysis in Arabidopsis
Source: Front Plant Sci. 2021 Mar 4;12:638051. doi: 10.3389/fpls.2021.638051 (PMC7969724; doi:10.3389/fpls.2021.638051)
Supplement: Supplementary file 3 [file Presentation_3.PPTX]

## Slide 1
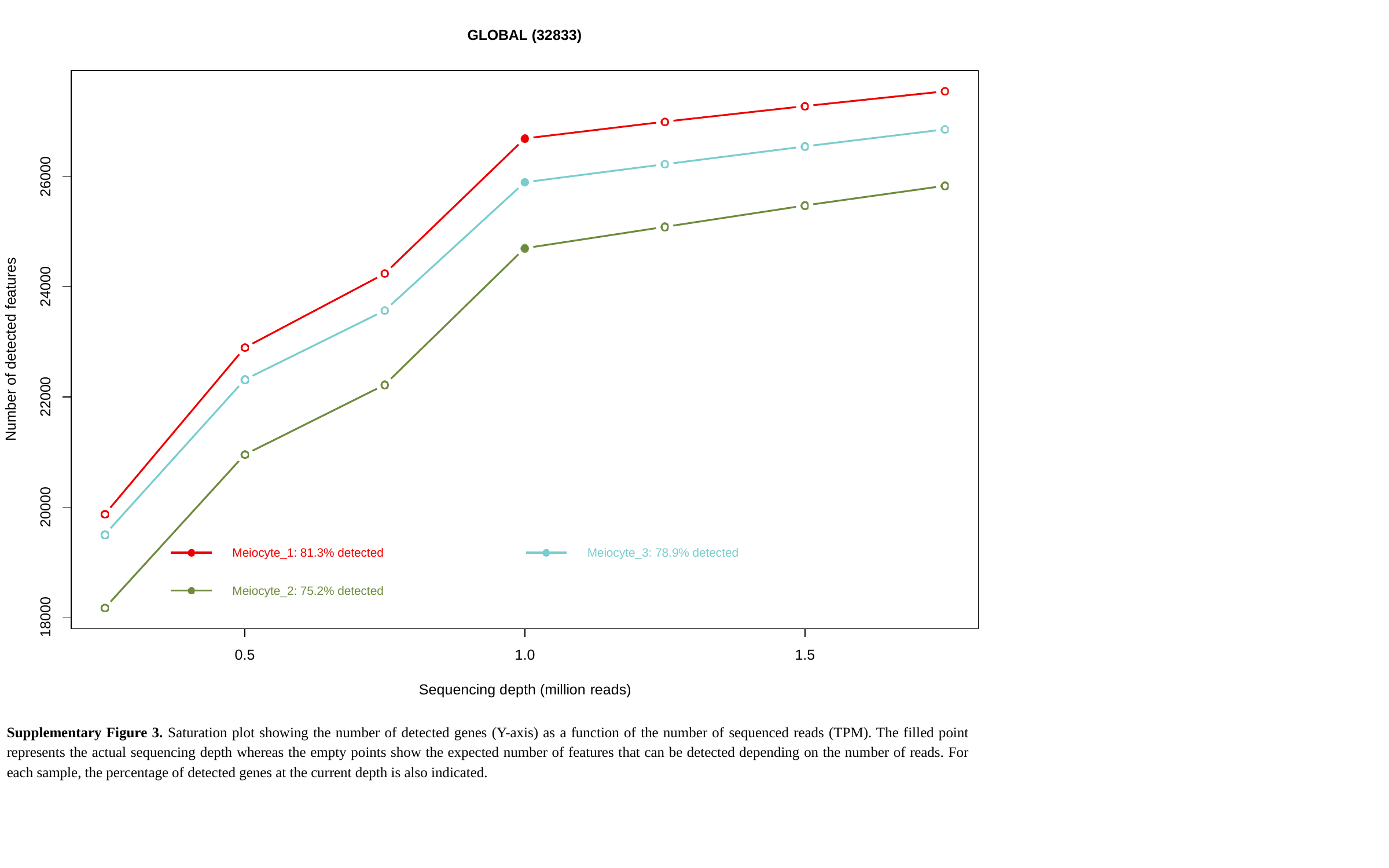

GLOBAL (32833)
26000
Number of detected features
24000
22000
20000
Meiocyte_1: 81.3% detected
Meiocyte_3: 78.9% detected
Meiocyte_2: 75.2% detected
18000
0.5
1.0
1.5
Sequencing depth (million reads)
Supplementary Figure 3. Saturation plot showing the number of detected genes (Y-axis) as a function of the number of sequenced reads (TPM). The filled point represents the actual sequencing depth whereas the empty points show the expected number of features that can be detected depending on the number of reads. For each sample, the percentage of detected genes at the current depth is also indicated.
