# Supplementary material for: Meiocyte Isolation by INTACT and Meiotic Transcriptome Analysis in Arabidopsis
Source: Front Plant Sci. 2021 Mar 4;12:638051. doi: 10.3389/fpls.2021.638051 (PMC7969724; doi:10.3389/fpls.2021.638051)
Supplement: Supplementary file 4 [file Presentation_4.PPTX]

## Slide 1
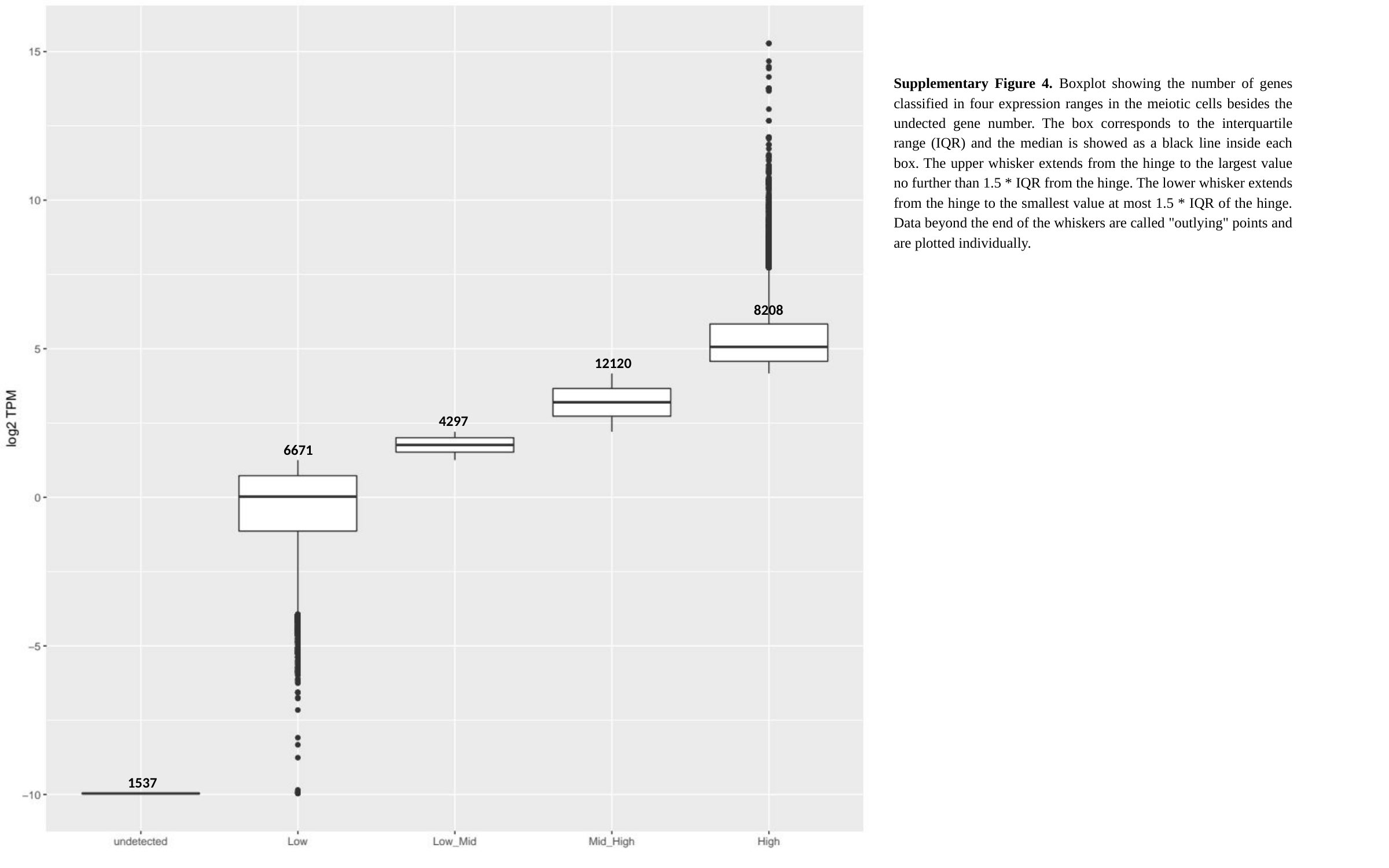

Supplementary Figure 4. Boxplot showing the number of genes classified in four expression ranges in the meiotic cells besides the undected gene number. The box corresponds to the interquartile range (IQR) and the median is showed as a black line inside each box. The upper whisker extends from the hinge to the largest value no further than 1.5 * IQR from the hinge. The lower whisker extends from the hinge to the smallest value at most 1.5 * IQR of the hinge. Data beyond the end of the whiskers are called "outlying" points and are plotted individually.
8208
12120
4297
6671
1537
