# Supplementary material for: Meiocyte Isolation by INTACT and Meiotic Transcriptome Analysis in Arabidopsis
Source: Front Plant Sci. 2021 Mar 4;12:638051. doi: 10.3389/fpls.2021.638051 (PMC7969724; doi:10.3389/fpls.2021.638051)
Supplement: Supplementary file 5 [file Presentation_5.PPTX]

## Slide 1
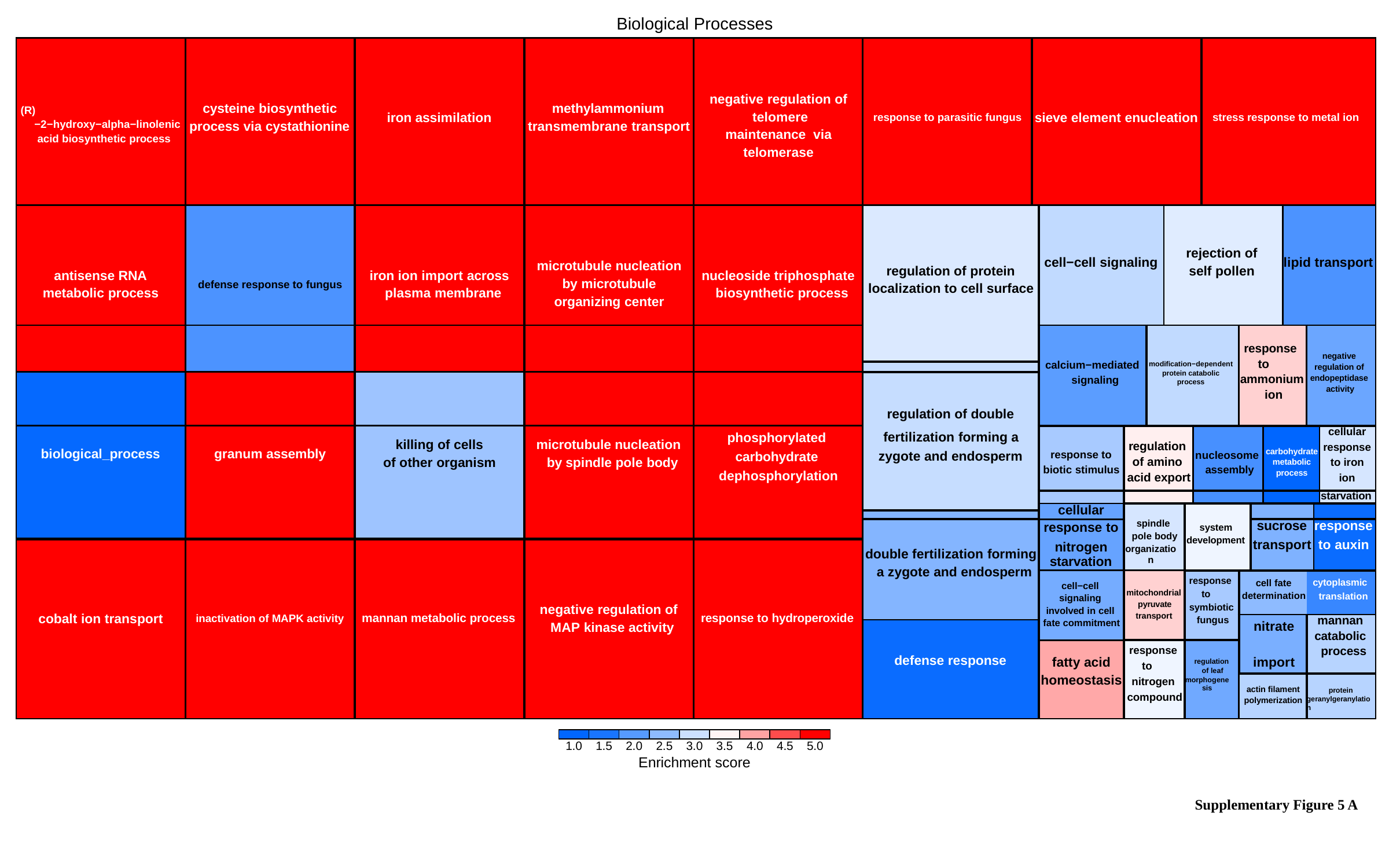

Biological Processes
| (R)−2−hydroxy−alpha−linolenic acid biosynthetic process | cysteine biosynthetic process via cystathionine | iron assimilation | methylammonium transmembrane transport | negative regulation of telomere maintenance via telomerase | response to parasitic fungus | sieve element enucleation | | | | | | | stress response to metal ion | | | | | | | |
| --- | --- | --- | --- | --- | --- | --- | --- | --- | --- | --- | --- | --- | --- | --- | --- | --- | --- | --- | --- | --- |
| antisense RNA metabolic process | defense response to fungus | iron ion import across plasma membrane | microtubule nucleation by microtubule organizing center | nucleoside triphosphate biosynthetic process | regulation of protein localization to cell surface | | cell−cell signaling | | | rejection of self pollen | | | | | | | lipid transport | | | |
| | | | | | | | calcium−mediated signaling | | modification−dependent protein catabolic process | | | | | response to ammonium ion | | | | negative regulation of endopeptidase activity | | |
| | | | | | | | | | | | | | | | | | | | | |
| | | | | | regulation of double | | | | | | | | | | | | | | | |
| biological\_process | granum assembly | killing of cells of other organism | microtubule nucleation by spindle pole body | phosphorylated carbohydrate dephosphorylation | fertilization forming a zygote and endosperm | | response to biotic stimulus | regulation of amino acid export | | | | nucleosome assembly | | | | carbohydrate metabolic process | | | | cellular response to iron ion |
| | | | | | | | | | | | | | | | | | | | | starvation |
| | | | | | | | cellular | spindle pole body organization | | | system development | | | | | | | | | |
| | | | | | | | | | | | | | | | | | | | | |
| | | | | | | | response to | | | | | | | | sucrose | | | | response | |
| cobalt ion transport | inactivation of MAPK activity | mannan metabolic process | negative regulation of MAP kinase activity | response to hydroperoxide | double fertilization forming a zygote and endosperm | | nitrogen starvation | | | | | | | | transport | | | | to auxin | |
| | | | | | | | cell−cell signaling involved in cell fate commitment | mitochondrial pyruvate transport | | | response to symbiotic fungus | | | cell fate determination | | | | cytoplasmic translation | | |
| | | | | | | | | | | | | | | nitrate import | | | | mannan catabolic process | | |
| | | | | | defense response | | | | | | | | | | | | | | | |
| | | | | | | | fatty acid homeostasis | response to nitrogen compound | | | regulation of leaf morphogenesis | | | | | | | | | |
| | | | | | | | | | | | | | | actin filament polymerization | | | | protein geranylgeranylation | | |
1.0	1.5	2.0	2.5	3.0	3.5	4.0	4.5	5.0
Enrichment score
Supplementary Figure 5 A

## Slide 2
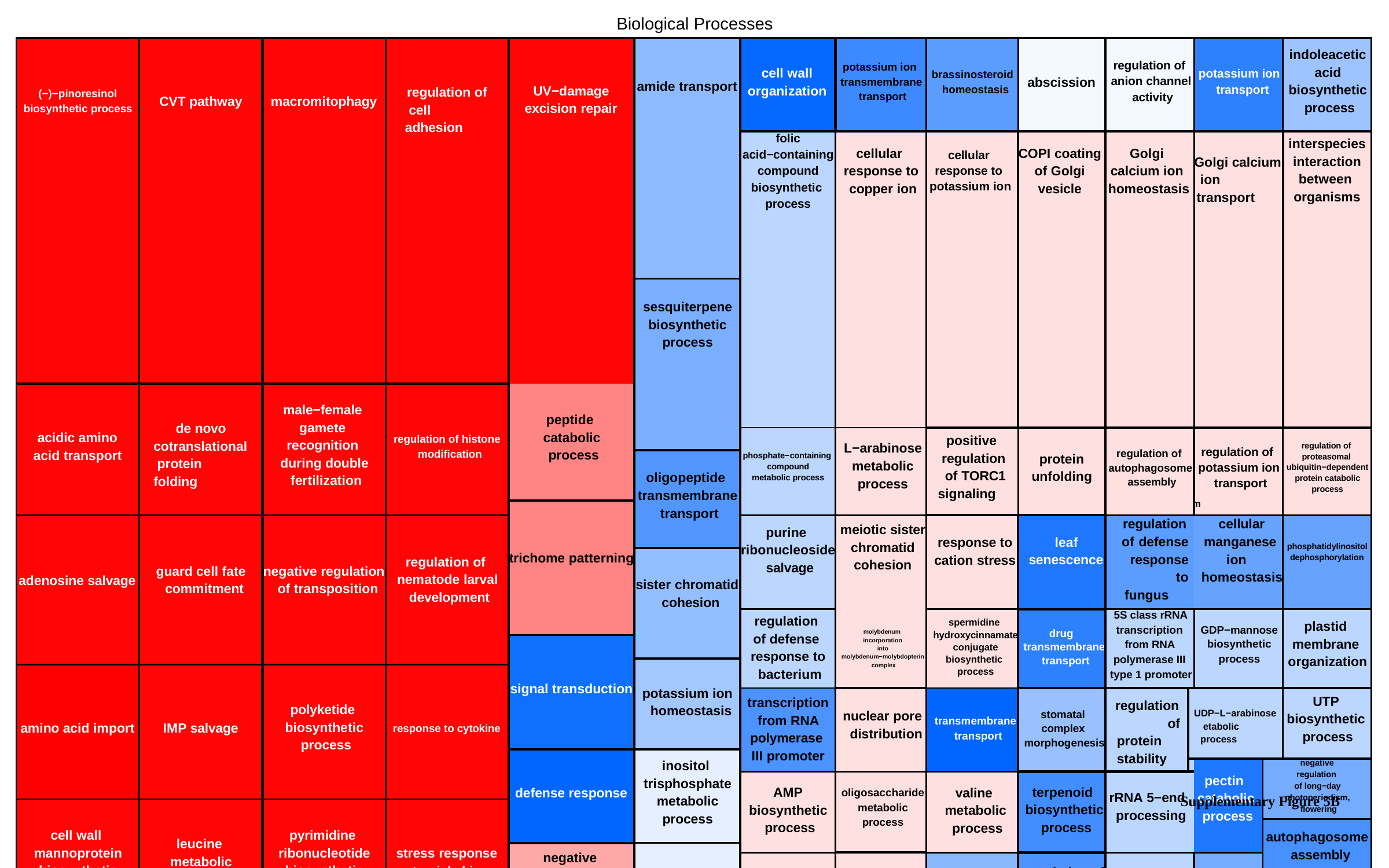

Biological Processes
| (−)−pinoresinol biosynthetic process | CVT pathway | macromitophagy | regulation of cell adhesion | UV−damage excision repair | amide transport | cell wall organization | potassium ion transmembrane transport | brassinosteroid homeostasis | abscission | regulation of anion channel activity | | potassium ion transport | | indoleacetic acid biosynthetic process |
| --- | --- | --- | --- | --- | --- | --- | --- | --- | --- | --- | --- | --- | --- | --- |
| | | | | | | folic acid−containing compound biosynthetic process | cellular response to copper ion | cellular response to potassium ion | COPI coating of Golgi vesicle | Golgi calcium ion homeostasis | | Golgi calcium ion transport | | interspecies interaction between organisms |
| | | | | | sesquiterpene biosynthetic process | | | | | | | | | |
| acidic amino acid transport | de novo cotranslational protein folding | male−female gamete recognition during double fertilization | regulation of histone modification | peptide catabolic process | | | | | | | | | | |
| | | | | | | phosphate−containing compound metabolic process | L−arabinose metabolic process | positive regulation of TORC1 signaling | protein unfolding | regulation of autophagosome assembly | | regulation of potassium ion transport | | regulation of proteasomal ubiquitin−dependent protein catabolic process |
| | | | | | oligopeptide transmembrane transport | | | | | | | | | |
| | | | | trichome patterning | | | | | | | | | | |
| adenosine salvage | guard cell fate commitment | negative regulation of transposition | regulation of nematode larval development | | | purine ribonucleoside salvage | meiotic sister chromatid cohesion | response to cation stress | leaf senescence | regulation of defense response to fungus | | cellular manganese ion homeostasis | | phosphatidylinositol dephosphorylation |
| | | | | | sister chromatid cohesion | | | | | | | | | |
| | | | | | | regulation of defense response to bacterium | molybdenum incorporation into molybdenum−molybdopterin complex | spermidine hydroxycinnamate conjugate biosynthetic process | drug transmembrane transport | 5S class rRNA transcription from RNA polymerase III type 1 promoter | | GDP−mannose biosynthetic process | | plastid membrane organization |
| | | | | signal transduction | | | | | | | | | | |
| | | | | | potassium ion homeostasis | | | | | | | | | |
| amino acid import | IMP salvage | polyketide biosynthetic process | response to cytokine | | | | | | | | | | | |
| | | | | | | transcription from RNA polymerase III promoter | nuclear pore distribution | transmembrane transport | stomatal complex morphogenesis | regulation of protein stability | | UDP−L−arabinose etabolic process | | UTP biosynthetic process |
| | | | | defense response | inositol trisphosphate metabolic process | | | | | | | | | |
| | | | | | | | | | | | | pectin catabolic process | negative regulation of long−day photoperiodism, flowering | |
| | | | | | | AMP biosynthetic process | oligosaccharide metabolic process | valine metabolic process | terpenoid biosynthetic process | rRNA 5−end processing | | | | |
| cell wall mannoprotein biosynthetic process | leucine metabolic process | pyrimidine ribonucleotide biosynthetic process | stress response to nickel ion | | | | | | | | | | | |
| | | | | | | | | | | | | | autophagosome assembly | |
| | | | | negative regulation of posttranscriptional gene silencing | regulation of autophagy | | | | | | | | | |
| | | | | | | attachment of spindle microtubules to kinetochore involved in homologous chromosome segregation | pollen−pistil interaction | drought recovery | regulation of intracellular pH | transpiration | | mRNA transcription | | |
| | | | | | | | | | | | | | response to nematode | |
m
1 2 3 4 5 6 7
Enrichment score
Supplementary Figure 5B

## Slide 3
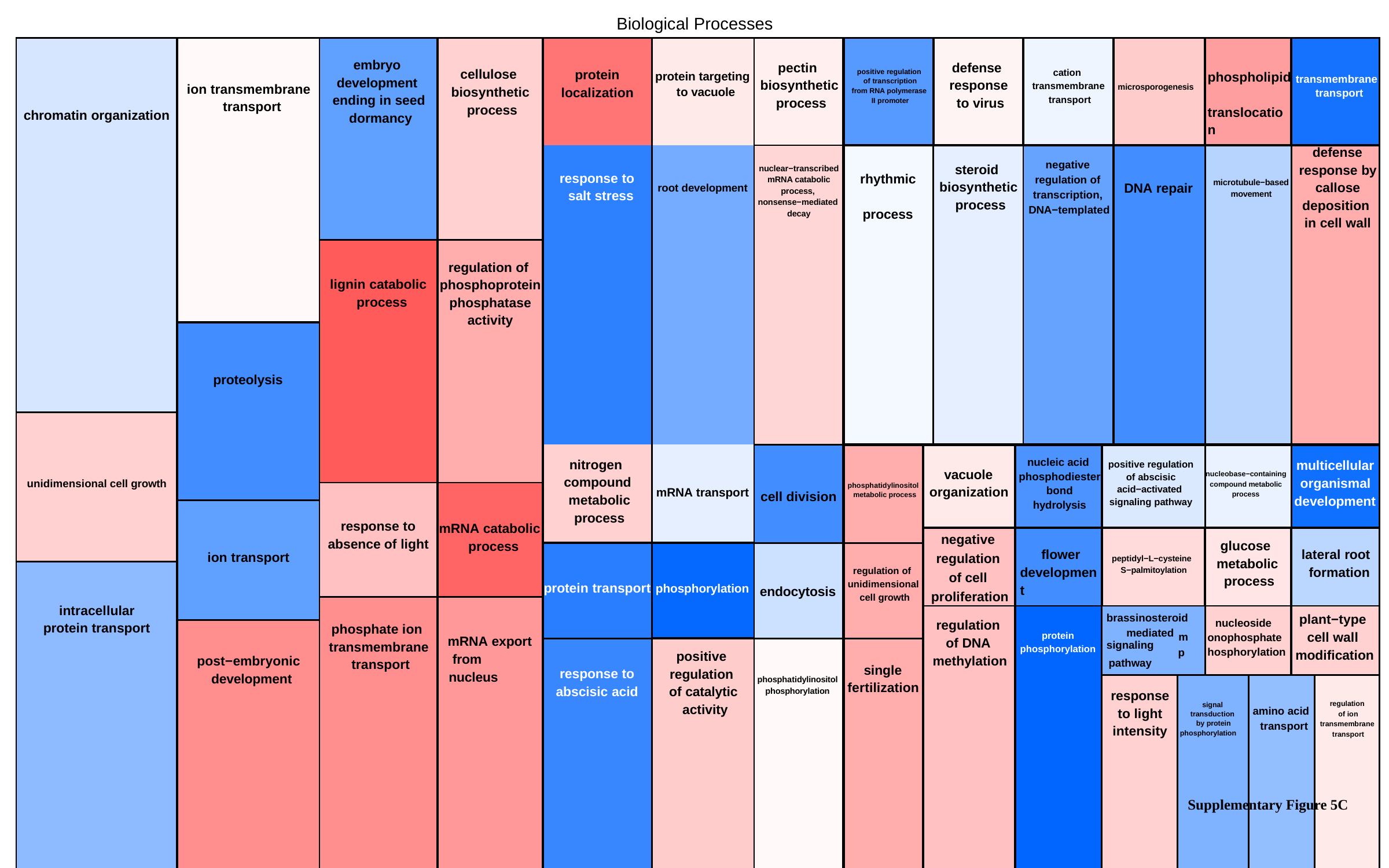

Biological Processes
| chromatin organization | ion transmembrane transport | embryo development ending in seed dormancy | cellulose biosynthetic process | protein localization | protein targeting to vacuole | pectin biosynthetic process | positive regulation of transcription from RNA polymerase II promoter | | defense response to virus | | cation transmembrane transport | | microsporogenesis | | phospholipid translocation | | | transmembrane transport | | |
| --- | --- | --- | --- | --- | --- | --- | --- | --- | --- | --- | --- | --- | --- | --- | --- | --- | --- | --- | --- | --- |
| | | | | response to salt stress | root development | nuclear−transcribed mRNA catabolic process, nonsense−mediated decay | rhythmic process | | steroid biosynthetic process | | negative regulation of transcription, DNA−templated | | DNA repair | | microtubule−based movement | | | defense response by callose deposition in cell wall | | |
| | | lignin catabolic process | regulation of phosphoprotein phosphatase activity | | | | | | | | | | | | | | | | | |
| | proteolysis | | | | | | | | | | | | | | | | | | | |
| unidimensional cell growth | | | | | | | | | | | | | | | | | | | | |
| | | | | nitrogen compound metabolic process | mRNA transport | cell division | phosphatidylinositol metabolic process | vacuole organization | | nucleic acid phosphodiester bond hydrolysis | | positive regulation of abscisic acid−activated signaling pathway | | | nucleobase−containing compound metabolic process | | | multicellular organismal development | | |
| | | response to absence of light | mRNA catabolic process | | | | | | | | | | | | | | | | | |
| | ion transport | | | | | | | | | | | | | | | | | | | |
| | | | | | | | | negative regulation of cell proliferation | | flower development | | peptidyl−L−cysteine S−palmitoylation | | | glucose metabolic process | | | lateral root formation | | |
| | | | | protein transport | phosphorylation | endocytosis | regulation of unidimensional cell growth | | | | | | | | | | | | | |
| intracellular protein transport | | | | | | | | | | | | | | | | | | | | |
| | | phosphate ion transmembrane transport | mRNA export from nucleus | | | | | | | | | | | | | | | | | |
| | | | | | | | | regulation of DNA methylation | | protein phosphorylation | | brassinosteroid mediated m signaling p pathway | | | nucleoside onophosphate hosphorylation | | | plant−type cell wall modification | | |
| | post−embryonic development | | | | | | | | | | | | | | | | | | | |
| | | | | response to abscisic acid | positive regulation of catalytic activity | phosphatidylinositol phosphorylation | single fertilization | | | | | | | | | | | | | |
| | | | | | | | | | | | | response to light intensity | | signal transduction by protein phosphorylation | | amino acid transport | | | regulation of ion transmembrane transport | |
| DNA demethylation | | | | | | | | vesicle transport along actin filament | | ATP hydrolysis coupled proton transport | | | | | | | | | | |
| | | vesicle−mediated transport plant−type primary cell wall biogenesis | leaf development protein deubiquitination | | | | | | | | | | | | | | | | | |
| | response to cadmium ion mRNA processing | | | | | | | | | | | | | | | | | | | |
| | | | | activation of GTPase activity RNA catabolic process | pollen development | circadian rhythm | xylan catabolic process | | | | | | | | | | | | | |
| | | | | | | | | positive regulation of GTPase activity | | pollen tube development | | actin filament−based movement | | photosynthesis, light harvesting in photosystem I | | | negative regulation of translation | | | protein catabolic process |
| regulation of cell shape | | | | | | | | | | | | | | | | | | | | |
| | | | | | protein N−linked glycosylation | chlorophyll biosynthetic process | protein targeting to membrane | | | | | | | | | | | | | |
| | | | | | | | | magnesium ion transmembrane transport | | DNA duplex unwinding | | magnesium ion transport | | sphingolipid metabolic process | | | | | | |
| | | | | | | | | | | | | | | | | | RNA phosphodiester bond hydrolysis | | | |
1.0	1.2	1.4	1.6	1.8	2.0	2.2
Enrichment score
Supplementary Figure 5C

## Slide 4
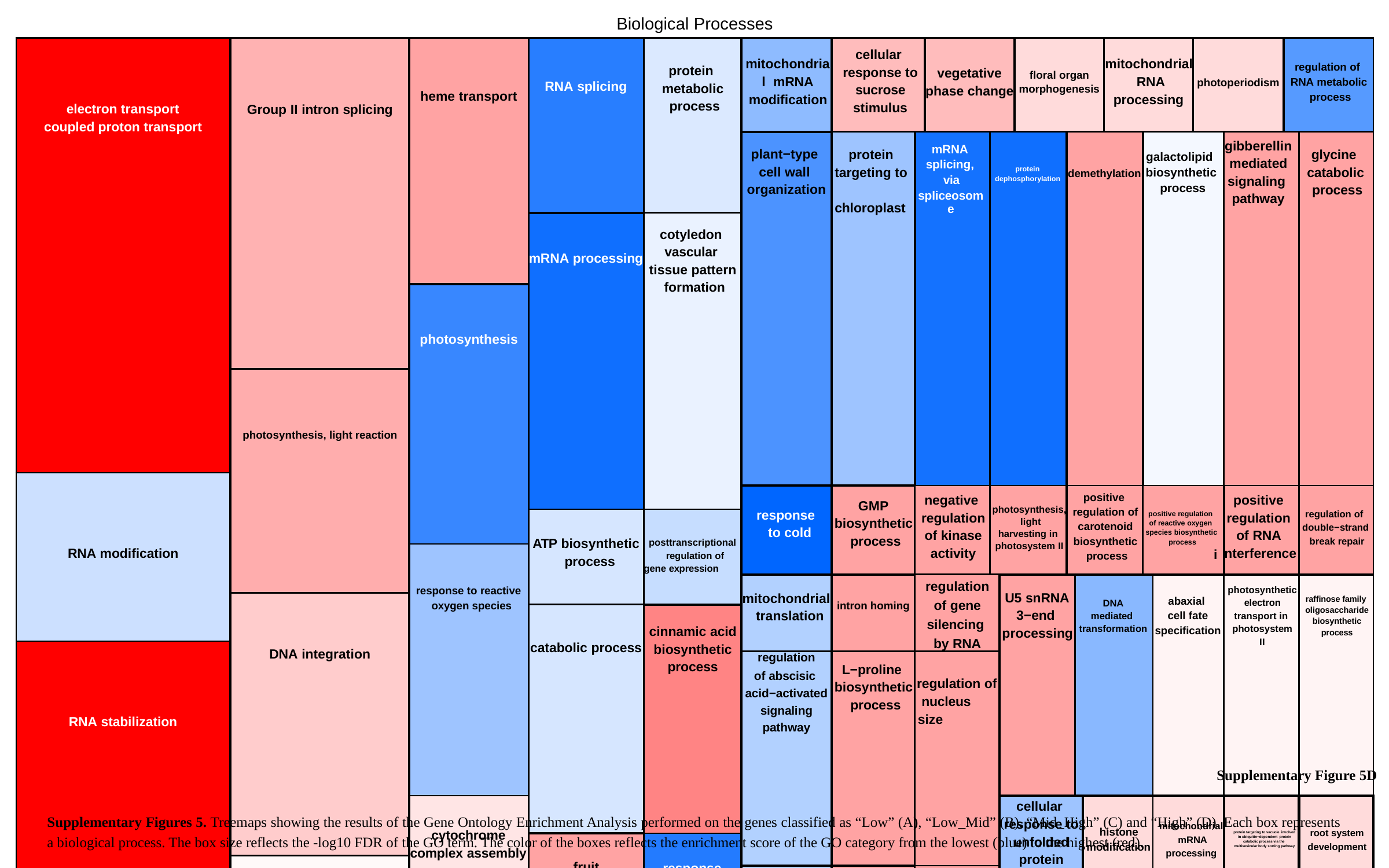

Biological Processes
| electron transport coupled proton transport | Group II intron splicing | heme transport | RNA splicing | protein metabolic process | mitochondrial mRNA modification | cellular response to sucrose stimulus | | vegetative phase change | | | floral organ morphogenesis | | | | mitochondrial RNA processing | | | | photoperiodism | | | | regulation of RNA metabolic process | | |
| --- | --- | --- | --- | --- | --- | --- | --- | --- | --- | --- | --- | --- | --- | --- | --- | --- | --- | --- | --- | --- | --- | --- | --- | --- | --- |
| | | | | | plant−type cell wall organization | protein targeting to chloroplast | mRNA splicing, via spliceosome | | protein dephosphorylation | | | demethylation | | | | galactolipid biosynthetic process | | | | gibberellin mediated signaling pathway | | | | glycine catabolic process | |
| | | | mRNA processing | cotyledon vascular tissue pattern formation | | | | | | | | | | | | | | | | | | | | | |
| | | photosynthesis | | | | | | | | | | | | | | | | | | | | | | | |
| | photosynthesis, light reaction | | | | | | | | | | | | | | | | | | | | | | | | |
| RNA modification | | | | | | | | | | | | | | | | | | | | | | | | | |
| | | | | | response to cold | GMP biosynthetic process | negative regulation of kinase activity | | photosynthesis, light harvesting in photosystem II | | | positive regulation of carotenoid biosynthetic process | | | | positive regulation of reactive oxygen species biosynthetic process i | | | | positive regulation of RNA nterference | | | | regulation of double−strand break repair | |
| | | | ATP biosynthetic process | posttranscriptional regulation of gene expression | | | | | | | | | | | | | | | | | | | | | |
| | | response to reactive oxygen species | | | | | | | | | | | | | | | | | | | | | | | |
| | | | | | mitochondrial translation | intron homing | regulation of gene silencing by RNA | | | U5 snRNA 3−end processing | | | DNA mediated transformation | | | | abaxial cell fate specification | | | photosynthetic electron transport in photosystem II | | | | raffinose family oligosaccharide biosynthetic process | |
| | DNA integration | | | | | | | | | | | | | | | | | | | | | | | | |
| | | | catabolic process | cinnamic acid biosynthetic process | | | | | | | | | | | | | | | | | | | | | |
| RNA stabilization | | | | | | | | | | | | | | | | | | | | | | | | | |
| | | | | | regulation of abscisic acid−activated signaling pathway | L−proline biosynthetic process | regulation of nucleus size | | | | | | | | | | | | | | | | | | |
| | | cytochrome complex assembly | | | | | | | | cellular response to unfolded protein | | | | histone modification | | | mitochondrial mRNA processing | | | protein targeting to vacuole involved in ubiquitin−dependent protein catabolic process via the multivesicular body sorting pathway | | | | root system development | |
| | | | fruit morphogenesis | response to heat | | | | | | | | | | | | | | | | | | | | | |
| | ATP synthesis coupled proton transport | | | | | | | | | | | | | | | | | | | | | | | | |
| | | | | | respiratory electron transport chain | mRNA modification | tRNA wobble adenosine to inosine editing | | | | | | | | | | | | | | | | | | |
| | | | | | | | | | | protein refolding | | | | | | | | | | | | | | | |
| | | | | | | | | | | | | | | tRNA thio−modification | | | | mRNA cis splicing, via spliceosome | | | RNA−dependent DNA replication | | | ribosome biogenesis | |
| | | protein folding | | | | | | | | | | | | | | | | | | | | | | | |
| | | | cellular protein modification process | nucleus organization | | | | | | | | | | | | | | | | | | | | | |
| translation | | | | | L−phenylalanine catabolic process | multivesicular body sorting pathway | tRNA wobble position uridine thiolation | | | RNA metabolic process | | | | | | | | | | | | | | | |
| | | | | | | | | | | | | | | chaperone mediated protein folding requiring cofactor | | | | | | | | | | | |
| | | | | | | | | | | | | | | | | | | cell redox homeostasis | | | | cytochrome b6f complex assembly | | | protein maturation by iron−sulfur cluster transfer |
| | ATP synthesis coupled electron transport | | | | | | | | | | | | | | | | | | | | | | | | |
| | | aerobic respiration | | | | | | | | | | | | | | | | | | | | | | | |
| | | | photosynthetic electron transport chain | regulation of seed growth | | | | | | | | | | | | | | | | | | | | | |
| | | | | | phloem transport | NADH oxidation | U1 snRNA 3−end processing | | | glycine decarboxylation via glycine cleavage system | | | | | | | | | | | | | | | |
| | | | | | | | | | | | | | | thylakoid membrane organization | | | | response to high light intensity | | | | | | | |
| | | | | | | | | | | | | | | | | | | | | | | rRNA catabolic process | | | |
1.5	2.0	2.5	3.0	3.5	4.0
Enrichment score
Supplementary Figure 5D
Supplementary Figures 5. Treemaps showing the results of the Gene Ontology Enrichment Analysis performed on the genes classified as “Low” (A), “Low_Mid” (B), “Mid_High” (C) and “High” (D). Each box represents a biological process. The box size reflects the -log10 FDR of the GO term. The color of the boxes reflects the enrichment score of the GO category from the lowest (blue) to the highest (red).
