# Supplementary material for: Meiocyte Isolation by INTACT and Meiotic Transcriptome Analysis in Arabidopsis
Source: Front Plant Sci. 2021 Mar 4;12:638051. doi: 10.3389/fpls.2021.638051 (PMC7969724; doi:10.3389/fpls.2021.638051)
Supplement: Supplementary file 6 [file Presentation_6.PPTX]

## Slide 1
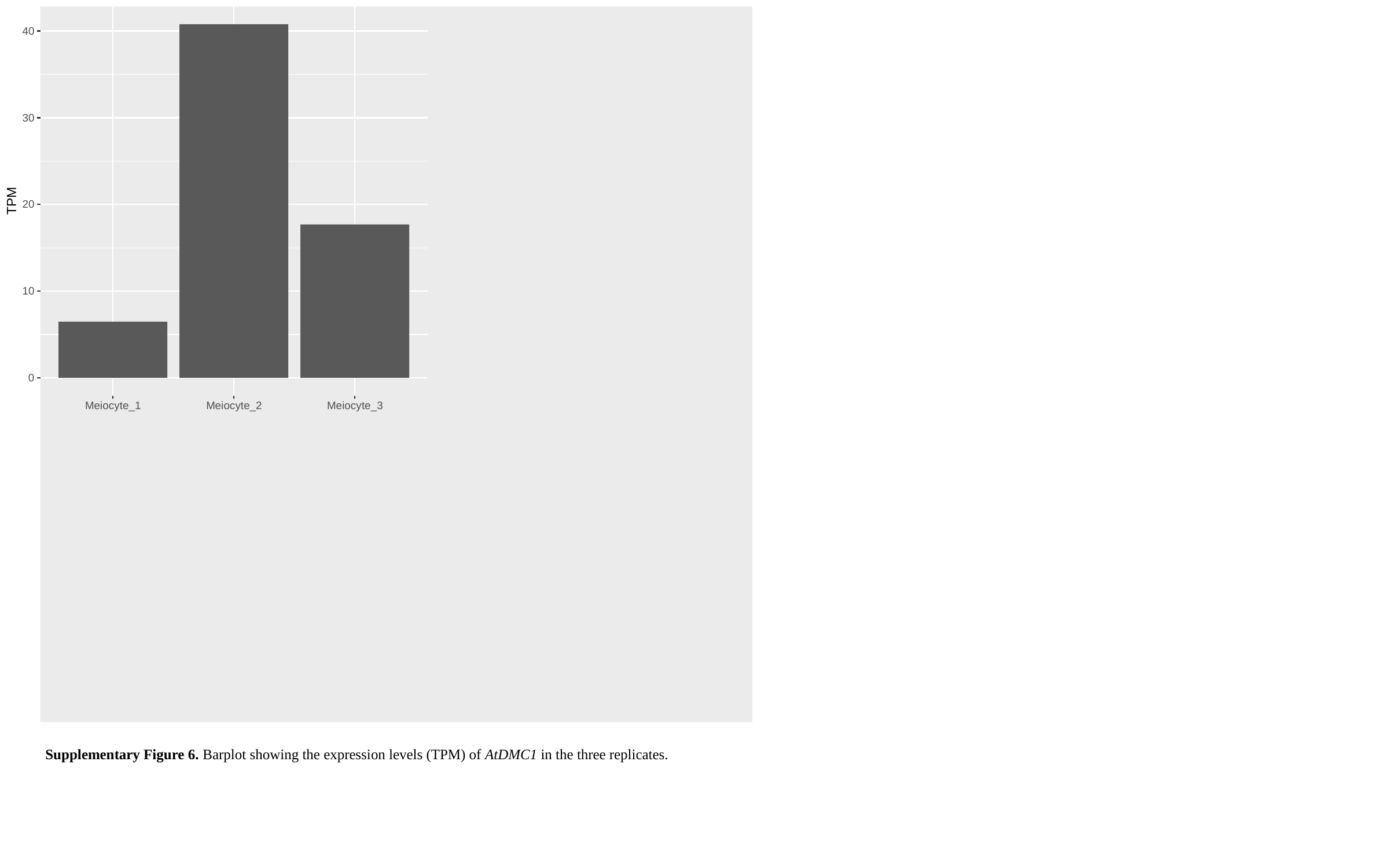

40
30
TPM
20
10
0
Meiocyte_1
Meiocyte_2
Meiocyte_3
Supplementary Figure 6. Barplot showing the expression levels (TPM) of AtDMC1 in the three replicates.
