# Supplementary material for: Meiocyte Isolation by INTACT and Meiotic Transcriptome Analysis in Arabidopsis
Source: Front Plant Sci. 2021 Mar 4;12:638051. doi: 10.3389/fpls.2021.638051 (PMC7969724; doi:10.3389/fpls.2021.638051)
Supplement: Supplementary file 7 [file Table_1.DOCX]

**Supplementary Table 1. Primers for hygromicin resistance gene**

| **Primer name** | **Sequence (5’-3’)** |
| --- | --- |
| Hyg_bf1 | CTCCATACAAGCCAACCACG |
| Hyg_br1 | CGATTGCTGATCCCCATGTG |
