# Supplementary material for: Meiocyte Isolation by INTACT and Meiotic Transcriptome Analysis in Arabidopsis
Source: Front Plant Sci. 2021 Mar 4;12:638051. doi: 10.3389/fpls.2021.638051 (PMC7969724; doi:10.3389/fpls.2021.638051)
Supplement: Supplementary file 8 [file Table_2.DOCX]

**Supplementary Table 2. Primers for BirA expression**

| **Primer name** | **Sequence (5’-3’)** |
| --- | --- |
| ACT2p:BirA-fw | ACAAAATTTAGAACGAACTTAATG |
| ACT2p:BirA-rev | GAATTCGGATATCGGTCG­AC |
| APT1-for | ATTTGTTCCCATGAGGAAGCC |
| APT1-rev | CACCTACGTGCATCTCAATCGT |
