# Supplementary material for: Meiocyte Isolation by INTACT and Meiotic Transcriptome Analysis in Arabidopsis
Source: Front Plant Sci. 2021 Mar 4;12:638051. doi: 10.3389/fpls.2021.638051 (PMC7969724; doi:10.3389/fpls.2021.638051)
Supplement: Supplementary file 9 [file Table_3.DOCX]

**Supplementary Table 3. Primers used for GATEWAY cloning strategy**

| **Primer name** | **Sequence (5’-3’)** | **Function** |
| --- | --- | --- |
| DMC1-ATT-B4 | GGGGACAACTTTGTATAGAAAAGTTGCTCAGCTATGAGATTACTCG | Cloning of *AtDMC1* promoter |
| DMC1-ATT-B1R | GGGGACTGCTTTTTTGTACAAACTTGCTTCTCGCTCTAAGACTCT | Cloning of *AtDMC1* promoter |
| NTF-ATT-B1 | GGGGACAAGTTTGTACAAAAAAGCAGGCTTAGATCATTCAGCGAAAACC | Cloning of *NTF* |
| NTF-ATT-B2 | GGGGACCACTTTGTACAAGAAAGCTGGGTATTCAAGATCCACCAGTAT | Cloning of *NTF* |
