# Supplementary material for: Meiocyte Isolation by INTACT and Meiotic Transcriptome Analysis in Arabidopsis
Source: Front Plant Sci. 2021 Mar 4;12:638051. doi: 10.3389/fpls.2021.638051 (PMC7969724; doi:10.3389/fpls.2021.638051)
Supplement: Supplementary file 19 [file Table_13.DOCX]

**Supplementary Table 13.** Meiosis-specific genes with established roles in prophase I identified in Mid/High and High expression classes of our dataset that can be used to build more INTACT lines.

| Locus | Gene | Expression Class |
| --- | --- | --- |
| AT1G67370 | ASY1 | Mid/High |
| AT4G35520 | MLH3 | High |
| AT4G17380 | MSH4 | Mid/High |
| AT1G10710 | PHS1 | Mid/High |
| AT5G48390 | ZYP4 | High |
| AT1G14750 | SDS | Mid/High |
